# In silico analysis to identify miR-1271-5p/PLCB4 (phospholipase C Beta 4) axis mediated oxaliplatin resistance in metastatic colorectal cancer

**DOI:** 10.1038/s41598-023-31331-2

**Published:** 2023-03-16

**Authors:** Cheng-Chin Lee, Ai-Wei Lee, Po-Li Wei, Yi-Shin Liu, Yu-Jia Chang, Chien-Yu Huang

**Affiliations:** 1 Graduate Institute of Medical Sciences, College of Medicine, Taipei Medical University, Taipei, Taiwan, ROC; 2 Department of Anatomy and Cell Biology, School of Medicine, College of Medicine, Taipei Medical University, Taipei, Taiwan, ROC; 3 Department of Surgery, School of Medicine, College of Medicine, Taipei Medical University, Taipei, Taiwan, ROC; 4 Division of Colorectal Surgery, Department of Surgery, Taipei Medical University Hospital, Taipei Medical University, Taipei, Taiwan, ROC; 5 Cancer Research Center and Translational Laboratory, Taipei Medical University Hospital, Taipei Medical University, Taipei, Taiwan, ROC; 6 Graduate Institute of Cancer Biology and Drug Discovery, Taipei Medical University, Taipei, Taiwan, ROC; 7 Graduate Institute of Clinical Medicine, College of Medicine, Taipei Medical University, Taipei, Taiwan, ROC; 8 Cell Physiology and Molecular Image Research Center, Wan Fang Hospital, Taipei Medical University, Taipei, Taiwan, ROC; 9 Department of Pathology, Wan Fang Hospital, Taipei Medical University, Taipei, Taiwan, ROC; 10 School of Medicine, National Tsing Hua University, Hsinchu, 300044 Taiwan, ROC; 11 Institute of Molecular and Cellular Biology, National Tsing Hua University, Hsinchu, 300044 Taiwan, ROC

**Keywords:** Cancer, Computational biology and bioinformatics

## Abstract

Oxaliplatin (OXA) is the first-line chemotherapy drug for metastatic colorectal cancer (mCRC), and the emergence of drug resistance is a major clinical challenge. Although there have been numerous studies on OXA resistance, but its underlying molecular mechanisms are still unclear. This study aims to identify key regulatory genes and pathways associated with OXA resistance. The Gene Expression Omnibus (GEO) GSE42387 dataset containing gene expression profiles of parental and OXA-resistant LoVo cells was applied to explore potential targets. GEO2R, STRING, CytoNCA (a plug-in of Cytoscape), and DAVID were used to analyze differentially expressed genes (DEGs), protein–protein interactions (PPIs), hub genes in PPIs, and gene ontology (GO)/Kyoto Encyclopedia of Genes and Genomes (KEGG) enrichment analysis. R2 online platform was used to run a survival analysis of validated hub genes enriched in KEGG pathways. The ENCORI database predicted microRNAs for candidate genes. A survival analysis of those genes was performed, and validated using the OncoLnc database. In addition, the 'clusterProfiler' package in R was used to perform gene set enrichment analysis (GSEA). We identified 395 DEGs, among which 155 were upregulated and 240 were downregulated. In total, 95 DEGs were screened as hub genes after constructing the PPI networks. Twelve GO terms and three KEGG pathways (steroid hormone biosynthesis, malaria, and pathways in cancer) were identified as being significant in the enrichment analysis of hub genes. Twenty-one hub genes enriched in KEGG pathways were defined as key genes. Among them *AKT3*, phospholipase C Beta 4 (*PLCB4*), and *TGFB1* were identified as OXA-resistance genes through the survival analysis. High expressions of *AKT3* and *TGFB1* were each associated with a poor prognosis, and lower expression of *PLCB4* was correlated with worse survival. Further, high levels of hsa-miR-1271-5p, which potentially targets *PLCB4*, were associated with poor overall survival in patients with CRC. Finally, we found that *PLCB4* low expression was associated with MAPK signaling pathway and VEGF signaling pathway in CRC. Our results demonstrated that hsa-miR-1271-5p/*PLCB4* in the pathway in cancer could be a new potential therapeutic target for mCRC with OXA resistance.

## Introduction

Colorectal cancer (CRC) is the third most common malignant disease worldwide, with over 1.8 million newly diagnosed cases and about 881,000 deaths each year^1^. In clinical settings, approximately one-fourth to one-fifth of CRC patients are found to have metastasized cancer at the time of diagnosis, and about half of all patients eventually develop metastatic CRC (mCRC)^2^. At present, surgery combined with chemotherapy is the main therapeutic method for CRC^3^. Chemotherapy is used to shrink the tumor before surgery or prevent recurrence after surgery in patients without metastasis^4^ and is used to control cancer progression in patients with mCRC^5^. FOLFOX (5-fluorouracil (5-FU), leucovorin, and oxaliplatin (OXA)) and CAPEOX (OXA and capecitabine) are OXA-based regimens that are most commonly used as the first-line treatment for mCRC^6^; however, the response rate in mCRC is less than 50%^7^. Although targeted therapies such as bevacizumab, cetuximab, panitumumab, and regorafenib have been used with chemotherapeutic drugs in first-line settings in recent years, but the therapeutic effect is hardly satisfactory, and the overall survival (OS) of mCRC patients is only around 30 months^8,9^. Therefore, optimizing or developing more-effective treatment strategies for mCRC is urgently needed.

OXA is a third-generation platinum-based chemotherapeutic agent that is mainly used for CRC, gastric cancer, and pancreatic cancer^10–12^. This drug is a bifunctional alkylating agent which targets DNA, forms platinum–DNA adducts to block DNA replication and transcription, and thus leads to the death of tumor cells^13^. Although OXA has fewer side effects than its precursors such as cisplatin or carboplatin^14^, chemotherapy-induced peripheral neuropathy during treatment still limits the efficacy of therapy^15^. Another major therapeutic obstacle is the development of OXA resistance, which could be intrinsic or acquired^16,17^. The mechanism of OXA resistance is multifactorial and complicated^18^. In addition to the drug efflux pump (copper transporters, sole carrier transporters, and ATP-binding cassette transporters) that can expel drugs and reduce intracellular concentrations in cancer cells, there are other mechanisms, such as activation of DNA repair systems, inhibition of cell death, detoxification via glutathione, the emergence of cancer stem cells, the epithelial-mesenchymal transition (EMT), and epigenetic alterations^16,17,19,20^. Accumulating evidence suggests that microRNAs (miRNAs) play essential roles in tumorigenesis and contribute to the oxaliplatin resistance of CRC^21^. By identifying these resistance-associated miRNAs, there is an opportunity to develop new therapeutic strategies for enhancing the sensitivity of tumor cells to Oxaliplatin^22^. However, there have been many studies on OXA resistance, but the underlying mechanism is still not fully understood.

In order to reveal the possible molecular mechanism of acquired OXA resistance in mCRC, we investigated the gene expression profiles of the LoVo cell line (supraclavicular lymph node metastasis) and its acquired OXA-resistant subline from the GEO public database. The key genes and pathways involved in OXA resistance were identified by an integrated bioinformatics approach, and three genes were found to be significantly associated with a poor prognosis of CRC patients. Our study uncovered the potential mechanism of drug resistance and provides future research directions for improving the treatment effects of OXA in patients with mCRC.

## Methods

### Microarray data

**Figure 1:**
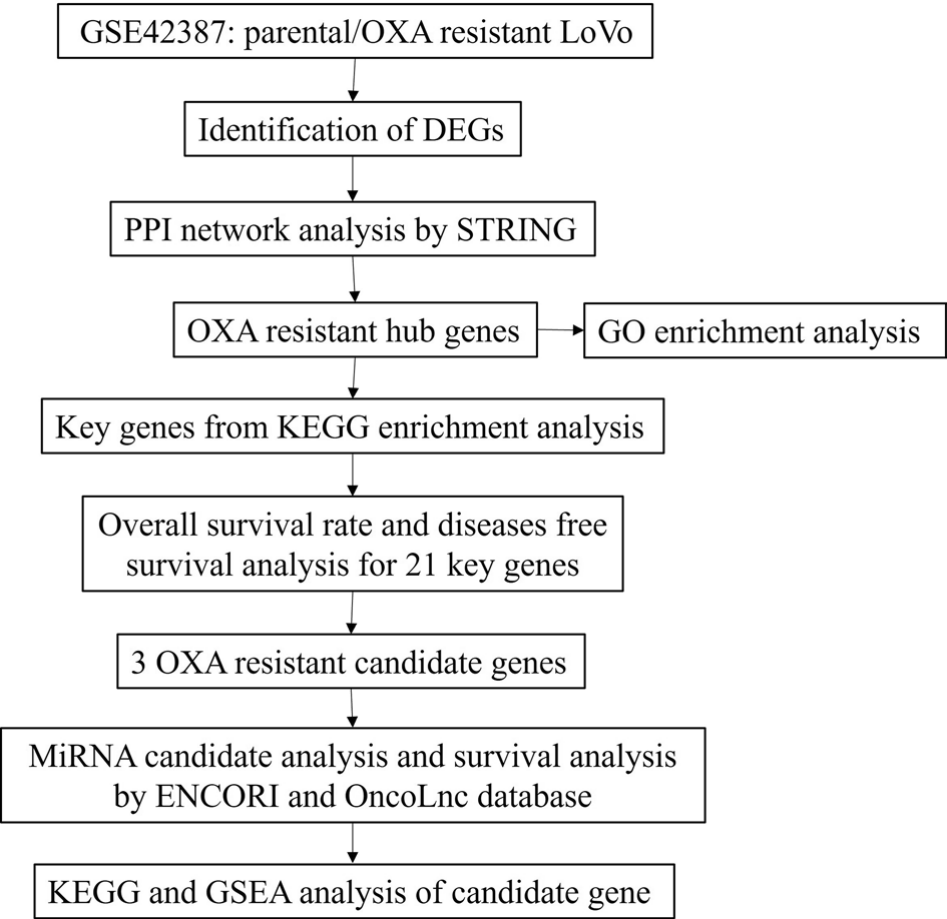
The flowchart of the bioinformatics analysis.

The gene expression profile of GSE42387, which is based on GPL16297 platform Agilent-014850 Whole Human Genome Microarray 4 × 44 K G4112F (Agilent Systematic Name, collapsed probe, version), was downloaded from the Gene Expression Omnibus (GEO) database (http://www.ncbi.nlm.nih.gov/geo/)^23^. The GSE42387 dataset contains expression patterns of parental and OXA-resistant LoVo cell lines. The resistant cell line was generated through exposure of the parental LoVo cell line to OXA in vitro with gradually increasing concentrations of OXA for a period of 265 days. Using the 50% inhibitory concentration (IC_50_) from an MTT assay to determine OXA sensitivity, the IC_50_ of the parental cell line was 1.1 ± 0.8 µM, and that of the drug-resistant cell line had increased to 15 ± 4.4 µM. After culturing the cell lines in a drug-free medium for 2–3 weeks, cells were harvested to extract total RNA in independent triplicates, followed by Agilent Human Gene Expression Microarrays (G4112F) for gene expression profiling^24^. The flowchart of the bioinformatics analysis is shown in Fig. 1.

### Data preprocessing and identification of differentially expressed genes (DEGs)

We selected the data of the parental and OXA-resistant LoVo cell lines from the GSE42387 dataset for the differential expression analysis by GEO2R friendly tool and recalculated the data^25^. The GEO2R online tool applies R language for GEO query and limma packages to examine gene expressions. The parental and OXA-resistant groups were separately selected to identify DEGs. A *t*-test and the Benjamini and Hochberg method were respectively used to calculate the *p* values and the false discovery rate (FDR)^26^. A *p* value of < 0.05 and |log[fold change (FC)]| of > 1 were set as DEG cutoff criteria. A volcano plot was created with the imageGP online tool (http://www.ehbio.com/ImageGP/), and a bidirectional hierarchical clustered heat map was generated using Tbtools software (https://github.com/CJ-Chen/TBtools)^27^.

### PPI network construction and hub gene screening

PPI networks were constructed by STRING (http://www.string-db.org/)^28^. This database provides known proteins and predicted protein interactions derived from four sources: genomic contexts, co-expressions, high-throughput experiments, and previous knowledge. A score of 0.7 (high confidence) was selected as the cutoff criterion. PPI pairs were introduced into Cytoscape software (vers. 3.8.2, http://www.cytoscape.org) and analyzed with the CytoNCA app in Cytoscape^29^. Hub genes (highly connected genes) were identified by calculating the degree value (number of lines connecting the genes) with a cutoff of ≥ 2.

### Enrichment analysis of hub genes and PLCB4 co-expression genes

The Database for Annotation, Visualization, and Integrated Discovery (DAVID, http://david.abcc.ncifcrf.gov/)^30^ was employed for functional enrichment analysis of hub genes using the Gene Ontology (GO) annotation (http://www.geneontology.org/)^31^ and Kyoto Encyclopedia of Genes and Genomes (KEGG; http://www.genome.jp/kegg/) pathway^32^. A *p* value of < 0.05 and an FDR value of < 0.25 were considered statistically significant. Hub genes enriched in KEGG pathways were regarded as key genes.

### Clinical validation of OXA-resistant genes

The R2: Genomics Analysis and Visualization Platform (http://r2.amc.nl) is a web-based tool that is freely available to the public, and it enables biomedical researchers who lack specialized training in bioinformatics to integrate, analyze, and visualize clinical and genomics data. A clinical assessment of key genes associated with OXA resistance was performed using this platform. Kaplan–Meier (KM) survival curves were drawn using "Tumor Colon (CMS)—Guinney—3232—custom—ccrcst1" for OS and relapse-free survival (RFS), "Tumor Colon—SieberSmith—355—MAS5.0—u133p2" for RFS, and "Tumor Colon—Smith—232—MAS5.0—u133p2" for disease-free survival (DFS). The cutoff modus was selected as “first_vs_last_quartile” to determine the threshold point, and *p* < 0.05 was considered the level of significance.

### Predicting micro (mi)RNAs for candidate genes

The Encyclopedia of RNA Interactomes (ENCORI) (http://starbase.sysu.edu.cn/; vers. 3.0) is an open-source platform that provides a series of miRNA-messenger (m)RNA prediction databases to explore miRNA–mRNA interactions. In the present study, the targeted miRNAs of candidate genes were defined according to the positive results of three miRNA-target predicting databases, including PITA, miRmap, and TargetScan. Furthermore, Cytoscape was used to visualize the miRNA-mRNA interaction network.

### Clinical examination of miRNAs

In order to assess clinical associations of predicted miRNAs, the ENCORI (http://starbase.sysu.edu.cn/; vers. 3.0) online tool was used to conduct an OS analysis for colon cancer patients. A *p* value of < 0.05 was considered to indicate a statistically significant result, which was further be verified by OncoLnc (https://www.oncolnc.org).

### Geneset enrichment analysis (GSEA) analysis

*PLCB4* differential expression on TCGA RNA-seq data was performed using DESeq2 with default parameters and Benjamini–Hochberg correction^33^, and then we conducted GSEA analysis with the curated gene sets (c2.cp.v7.2.symbols.gmt) using the R package “clusterprofiler” in R 3.6.3^34^. The visualizations were created in R using ggplot2 graphics package.

## Results

### Identification of DEGs and heat map clustering analysis

**Figure 2:**
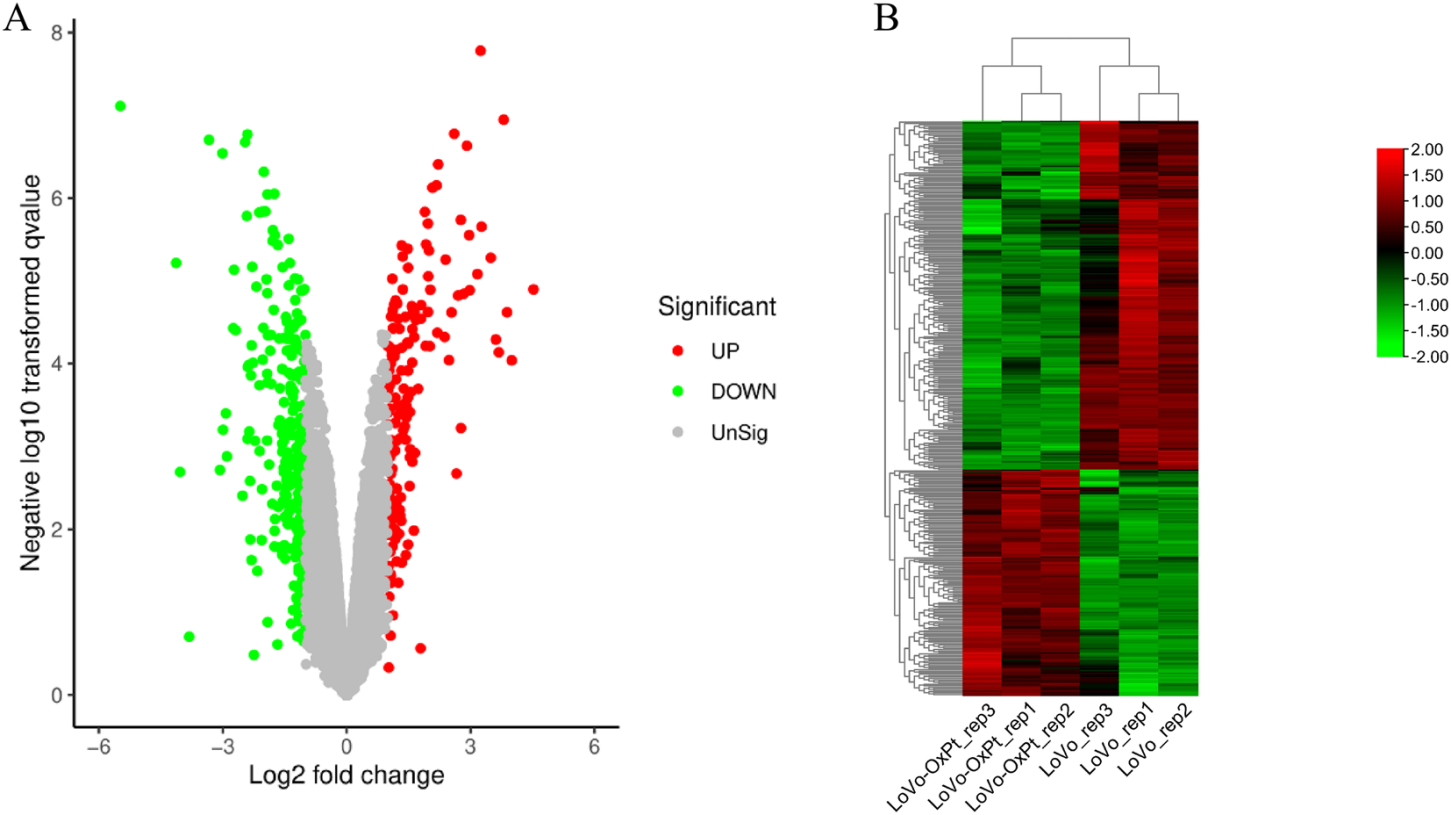
Identification of differentially expressed genes (DEGs). (**A**) Volcano plot of DEGs between parental LoVo and OXA resistant LoVo. (**B**) Heat map showing up-regulated and down-regulated differentially expressed genes (DEGs) in oxaliplatin-resistant LoVo cell line.

We analyzed the gene expression profiles from GSE42387. This dataset contains 27 samples from 3 different cell lines namely HCT116, HT29 and LoVo parental and oxaliplatin resistant sublines. Further in-depth bioinformatic analysis was conducted on LoVo parental and oxaliplatin resistant sub-lines. The GEO2R online tool was used to identify DEGs between parental and OXA-resistant LoVo cells. In total, 395 DEGs were predicted to be related to OXA resistance after calculation of log (FC) and *p* values; among these, 155 DEGs were upregulated and 240 DEGs were downregulated (Fig. 2A, Supplemental Fig. 1, Supplemental Table 1). Tbtools software was used to draw a heat map to obtain the bidirectional hierarchical clustering of the DEGs in which the upregulated and downregulated DEGs were summarized (Fig. 2B).

### PPI network and screening of hub genes

**Figure 3:**
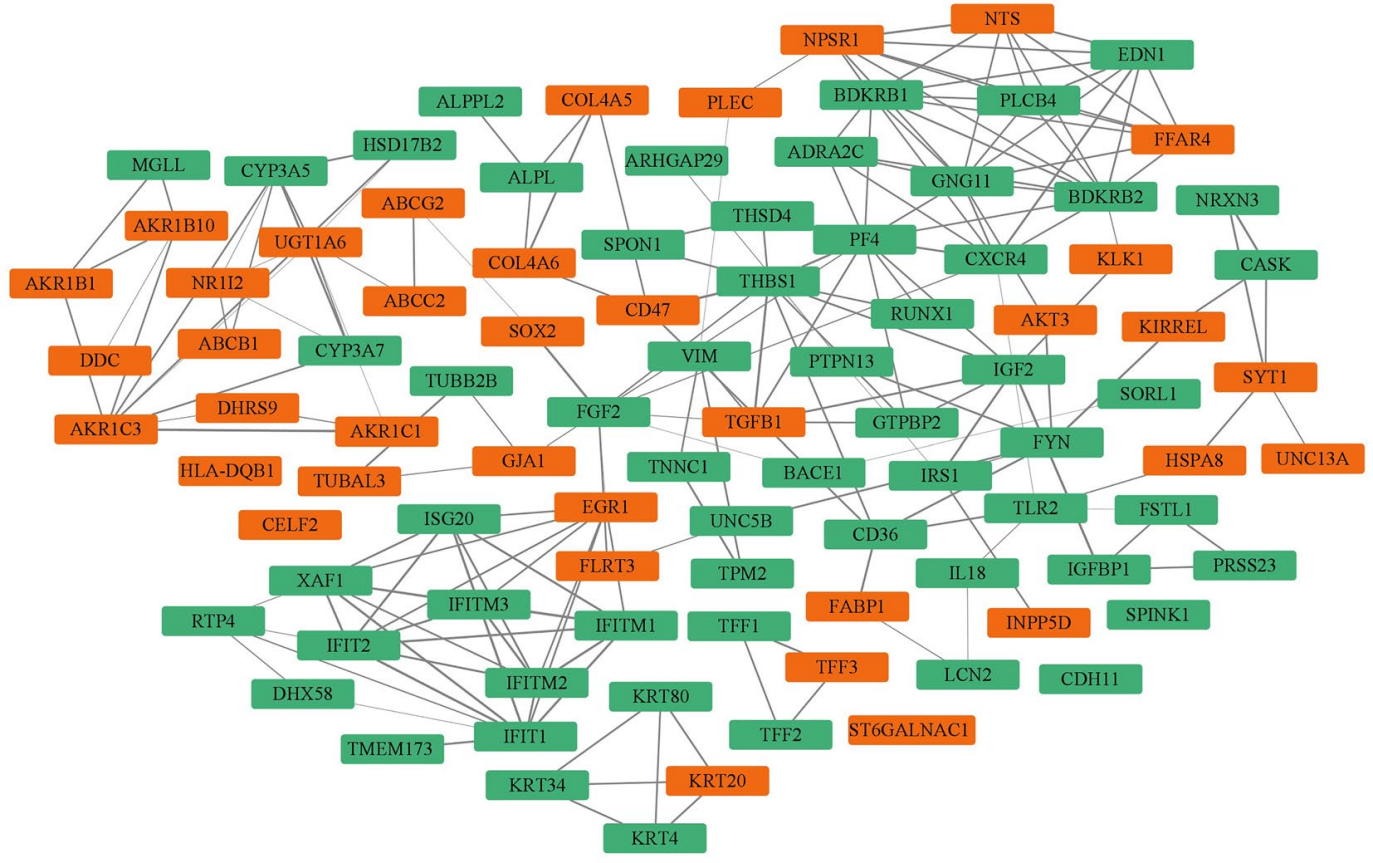
Protein–protein interaction (PPI) network of hub genes. The STRING network includes 95 nodes and 168 edges to represent 37 upregulated (Red rectangle) and 58 downregulated (Green rectangle) DEGs. The lines represent protein–protein interactions whereas the rectangles include the name of the protein.

The DEGs were loaded into the STRING database (https://string-db.org/) to obtain PPI pairs and then imported into Cytoscape software to identify hub genes. After the PPI analysis, 95 DEGs (37 upregulated and 58 downregulated genes) were identified as hub genes by the CytoNCA plugin (Table 1). A PPI network of 95 hub genes containing 95 nodes and 168 edges was obtained (Fig. 3).

**Table 1:** The Rank of hub genes. Hub genes are defined as which DEGs had interactions (Degree ≥ 2) in the protein–protein interaction (PPI) network.

No.	Hub genes	Degree	Betweenness	Closeness	logFC
1	*PF4*	12.0	1488.96	0.017	− 1.05
2	*FGF2*	11.0	6701.33	0.017	− 1.04
3	*BDKRB2*	11.0	312.75	0.017	− 1.56
4	*GNG11*	11.0	520.55	0.017	− 2.03
5	*IFIT1*	10.0	691.33	0.017	− 2.11
6	*THBS1*	10.0	2375.17	0.017	− 1.39
7	*BDKRB1*	10.0	212.42	0.017	− 1.04
8	*EGR1*	9.0	2302.00	0.017	1.04
9	*IGF2*	9.0	1679.00	0.017	− 2.42
10	*IFIT2*	8.0	67.33	0.017	− 1.43
11	*XAF1*	8.0	67.33	0.017	− 1.30
12	*EDN1*	8.0	92.94	0.017	− 1.66
13	*NPSR1*	8.0	176.00	0.017	1.98
14	*AKR1C3*	8.0	1234.33	0.016	1.80
15	*CXCR4*	8.0	2096.87	0.017	− 2.34
16	*ISG20*	7.0	0.00	0.017	− 1.28
17	*IFITM1*	7.0	0.00	0.017	− 1.03
18	*IFITM3*	7.0	0.00	0.017	− 1.13
19	*IFITM2*	7.0	0.00	0.017	− 1.12
20	*NTS*	7.0	0.00	0.017	3.88
21	*PLCB4*	7.0	0.00	0.017	− 1.19
22	*FFAR4*	7.0	0.00	0.017	1.59
23	*FYN*	7.0	1285.20	0.017	− 1.35
24	*UGT1A6*	7.0	2775.00	0.017	1.43
25	*CYP3A5*	7.0	332.00	0.016	− 1.29
26	*TGFB1*	6.0	1369.23	0.017	1.04
27	*IRS1*	5.0	1011.00	0.017	− 1.04
28	*TLR2*	5.0	2007.70	0.017	− 1.36
29	*CD36*	5.0	1283.13	0.017	− 1.66
30	*SYT1*	5.0	834.93	0.017	1.15
31	*ADRA2C*	5.0	0.00	0.017	− 1.03
32	*VIM*	4.0	900.50	0.017	− 2.02
33	*GTPBP2*	4.0	0.00	0.017	− 1.03
34	*HSPA8*	4.0	1288.93	0.017	1.07
35	*RTP4*	4.0	2.00	0.017	− 1.92
36	*CD47*	4.0	1020.00	0.017	1.37
37	*CYP3A7*	4.0	5.00	0.016	− 1.13
38	*AKR1B10*	4.0	523.00	0.016	3.68
39	*NR1I2*	4.0	94.67	0.016	1.21
40	*TFF3*	3.0	4.00	0.006	1.09
41	*TPM2*	3.0	212.00	0.017	− 1.03
42	*PLEC*	3.0	268.50	0.017	1.19
43	*KRT80*	3.0	0.00	0.006	− 2.90
44	*KRT4*	3.0	0.00	0.006	− 1.56
45	*KRT34*	3.0	0.00	0.006	− 2.36
46	*KRT20*	3.0	0.00	0.006	1.07
47	*HLA-DQB1*	3.0	6.00	0.006	1.10
48	*IGFBP1*	3.0	188.63	0.017	− 2.36
49	*FSTL1*	3.0	119.50	0.017	− 2.40
50	*GJA1*	3.0	420.00	0.017	1.12
51	*LCN2*	3.0	214.00	0.017	− 3.07
52	*UNC5B*	3.0	449.73	0.017	− 1.74
53	*CASK*	3.0	175.07	0.017	− 2.75
54	*PTPN13*	3.0	420.00	0.017	− 1.03
55	*COL4A6*	3.0	309.00	0.017	1.67
56	*COL4A5*	3.0	309.00	0.017	1.92
57	*ALPL*	3.0	420.00	0.017	− 1.17
58	*HSD17B2*	3.0	0.00	0.016	− 1.02
59	*DHRS9*	3.0	212.00	0.016	1.18
60	*AKR1C1*	3.0	187.00	0.016	4.00
61	*AKR1B1*	3.0	103.00	0.016	3.61
62	*ABCG2*	3.0	3238.00	0.017	1.02
63	*TNNC1*	2.0	0.00	0.017	− 1.45
64	*TFF2*	2.0	0.00	0.006	− 2.27
65	*TFF1*	2.0	0.00	0.006	− 2.19
66	*THSD4*	2.0	0.00	0.017	− 1.28
67	*SPON1*	2.0	0.00	0.017	− 1.10
68	*SPINK1*	2.0	2.00	0.006	− 1.75
69	*RUNX1*	2.0	0.00	0.017	− 1.05
70	*INPP5D*	2.0	212.00	0.017	1.54
71	*IL18*	2.0	187.73	0.017	− 1.42
72	*TMEM173*	2.0	212.00	0.017	− 1.09
73	*TUBAL3*	2.0	0.00	0.017	2.76
74	*TUBB2B*	2.0	0.00	0.017	− 1.17
75	*ST6GALNAC1*	2.0	2.00	0.006	1.36
76	*PRSS23*	2.0	0.00	0.017	− 1.90
77	*FLRT3*	2.0	429.47	0.017	1.20
78	*DHX58*	2.0	0.00	0.017	− 1.50
79	*CELF2*	2.0	2.00	0.006	1.17
80	*CDH11*	2.0	2.00	0.006	− 1.07
81	*FABP1*	2.0	228.27	0.017	4.52
82	*NRXN3*	2.0	0.00	0.017	− 1.38
83	*KIRREL*	2.0	309.07	0.017	1.26
84	*UNC13A*	2.0	212.00	0.016	1.01
85	*KLK1*	2.0	28.93	0.017	1.01
86	*SORL1*	2.0	212.00	0.017	− 1.01
87	*BACE1*	2.0	420.00	0.017	− 1.19
88	*ARHGAP29*	2.0	212.00	0.017	− 1.26
89	*ALPPL2*	2.0	212.00	0.016	− 2.32
90	*AKT3*	2.0	192.47	0.017	1.34
91	*DDC*	2.0	212.00	0.016	1.16
92	*MGLL*	2.0	0.00	0.016	− 1.15
93	*SOX2*	2.0	3344.00	0.017	1.43
94	*ABCC2*	2.0	2912.00	0.017	2.84
95	*ABCB1*	2.0	0.00	0.016	1.91

### GO terms and KEGG pathway enrichment analysis of hub genes

**Figure 4:**
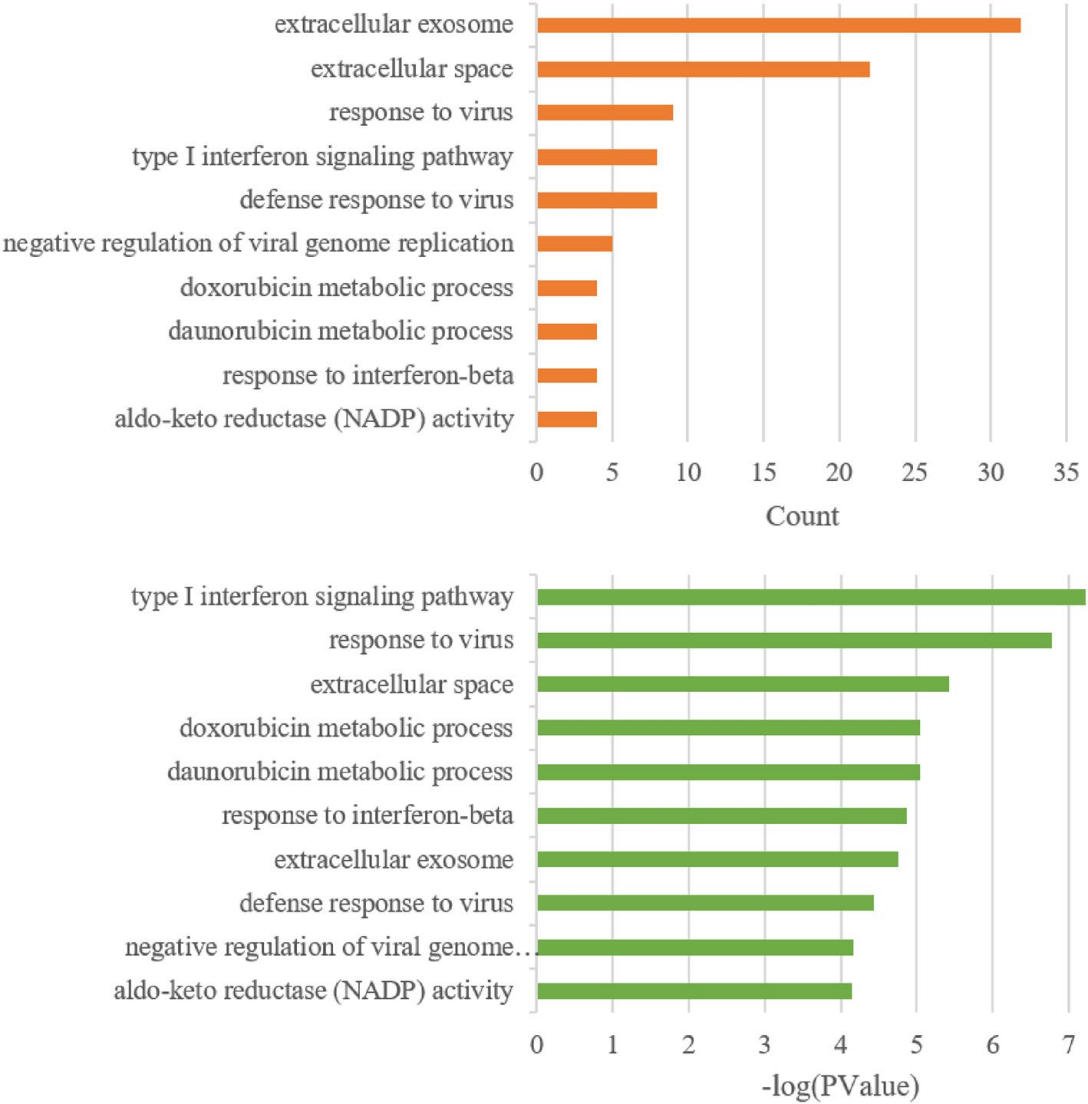
Top 10 Enriched gene ontologies (GO’s). The Y-axis stands for the GO biological process whereas the X-axis represents number of genes (upper) and significance (bottom).

**Figure 5:**
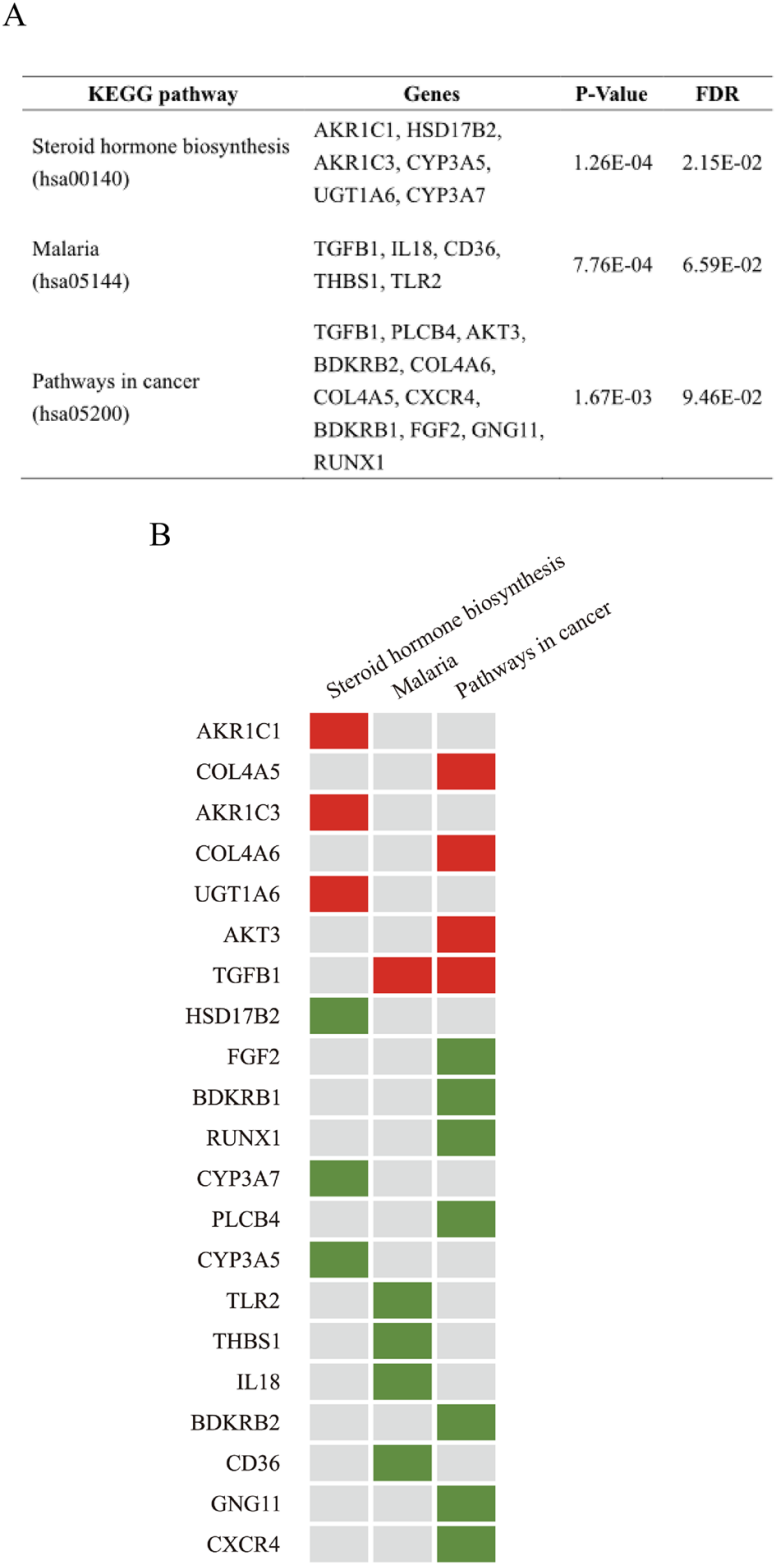
Enriched KEGG pathways. (**A**) List of significantly enriched KEGG pathways. (**B**) Hub genes that were enriched in KEGG pathways were considered key genes. Red indicates upregulated genes, and green indicates downregulated genes.

The 95 hub genes derived from the PPI network were applied to obtain GO terms and KEGG pathway enrichment analysis by the DAVID online tool. Enriched GO terms were divided into three categories, namely molecular functions (MFs), cellular component (CCs), and biological process (BPs). Results of the GO analysis demonstrated that hub genes were mainly enriched in BPs, including the type I interferon signaling pathway, response to viruses, daunorubicin metabolic processes, doxorubicin metabolic processes, responses to interferon-beta, defense responses to viruses, and negative regulation of viral genome replication. The CC analysis indicated that hub genes were significantly enriched in the extracellular space, extracellular exosomes, and plasma membranes. For MFs, hub genes were enriched in aldo–keto reductase (AKR) activity and indanol dehydrogenase activity (Fig. 4, Supplemental Table 2). The KEGG pathways, including steroid hormone biosynthesis (hsa00140), malaria (hsa05144), and pathways in cancer (hsa05200), were identified to be significant (Fig. 5A). There were seven upregulated and 14 downregulated hub genes enriched in KEGG pathways, and these 21 hub genes were regarded as key genes involved in OXA resistance (Fig. 5B).

### Clinical associations of key genes

**Figure 6:**
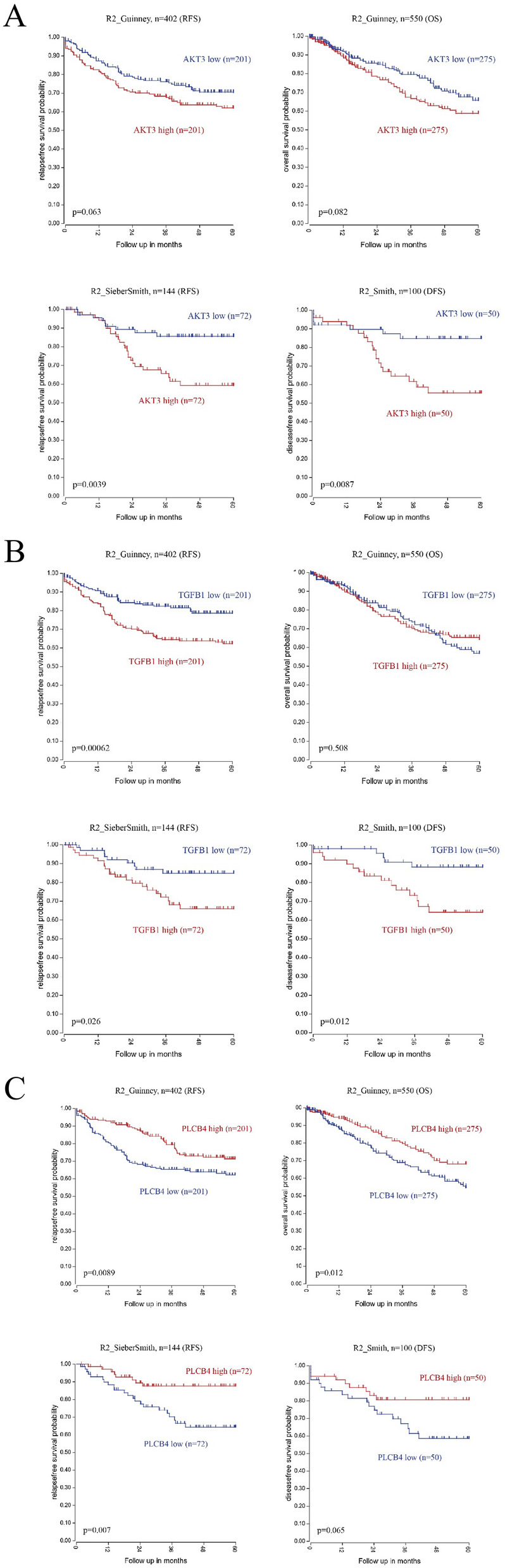
Kaplan Meier survival curves presenting the significant prognostic relationship between high and low expression of key genes by using the R2 platform. Up-regulated genes: (**A**) *AKT3* (**B**) *TGFB1*; down-regulated genes: (**C**) *PLCB4*.

Furthermore, we performed a survival analysis of all key genes by R2: Genomics Analysis and Visualization Platform (Tables 2, 3, Fig. 6, Supplemental Fig. 1). Notably, high *AKT3* and *TGFB1* expressions were significantly associated with poor outcomes from the SierberSmith and Smith datasets. High *TGFB1* expression was also significantly related to worse RFS from the Guinney dataset (Fig. 6A,B). Meanwhile, low *PLCB4* expression was significantly correlated with poor prognosis from the Guinney and SierberSmith datasets (Fig. 6C). This suggests that *AKT3*, *TGFB1*, and *PLCB4* could be potential candidates for OXA therapy.

**Table 2:** Survival analysis of up-regulated key genes.

Gene symbol	R2_Guinney, n = 402 (RFS)		R2_Guinney, n = 550 (OS)		R2_SieberSmith, n = 144 (RFS)		R2_Smith, n = 100 (RFS)	
	p value	Poor prognosis	p value	Poor prognosis	p value	Poor prognosis	p value	Poor prognosis
*AKR1C1*	0.00450*	High	0.19100	High	0.76200	Low	0.92600	Low
*COL4A5*	0.26000	High	0.25600	High	0.01300*	Low	0.10200	Low
*AKR1C3*	N/A	N/A	N/A	N/A	0.55400	Low	0.81900	Low
*COL4A6*	N/A	N/A	N/A	N/A	0.00130*	Low	0.00410*	Low
*UGT1A6*	N/A	N/A	N/A	N/A	0.27900	Low	0.16000	Low
*AKT3*	0.06300	High	0.08200	High	0.00390*	High	0.00870*	High
*TGFB1*	0.00062*	High	0.50800	Low	0.02600*	High	0.01200*	High

*p  < 0.05.

**Table 3:** Survival analysis of down-regulated key genes.

Gene symbol	R2_Guinney, n = 402 (RFS)		R2_Guinney, n = 550 (OS)		R2_SieberSmith, n = 144 (RFS)		R2_Smith, n = 100 (RFS)	
	p value	Poor prognosis	p value	Poor prognosis	p value	Poor prognosis	p value	Poor prognosis
*HSD17B2*	0.00680*	High	0.24800	High	0.72700	Low	0.28100	High
*FGF2*	0.30200	High	0.00620*	High	0.60500	Low	0.94300	Low
*BDKRB1*	0.00920*	High	0.01400*	High	0.86000	Low	0.95000	High
*RUNX1*	0.00100*	High	0.38500	High	0.03300*	High	0.01900*	High
*CYP3A7*	N/A	N/A	N/A	N/A	0.93100	Low	0.50700	High
*PLCB4*	0.00890*	Low	0.01200*	Low	0.00700*	Low	0.06500	Low
*CYP3A5*	0.82100	High	0.29300	High	0.46100	High	0.26800	High
*TLR2*	0.43000	High	0.96800	Low	0.63400	High	0.47300	High
*THBS1*	0.00790*	High	0.17000	High	0.00860*	High	0.51000	High
*IL18*	0.73600	Low	0.43200	Low	0.43800	High	0.69200	High
*BDKRB2*	0.51000	Low	0.88600	High	0.05200	Low	0.46100	Low
*CD36*	0.00250*	High	0.00019*	High	0.09700	High	0.00810*	High
*GNG11*	0.01100*	High	0.78300	High	0.10000	High	0.55300	High
*CXCR4*	0.00120*	High	0.84800	High	0.02200*	High	0.05000	High

*p  < 0.05.

### miRNA-candidate genes pairs

**Figure 7:**
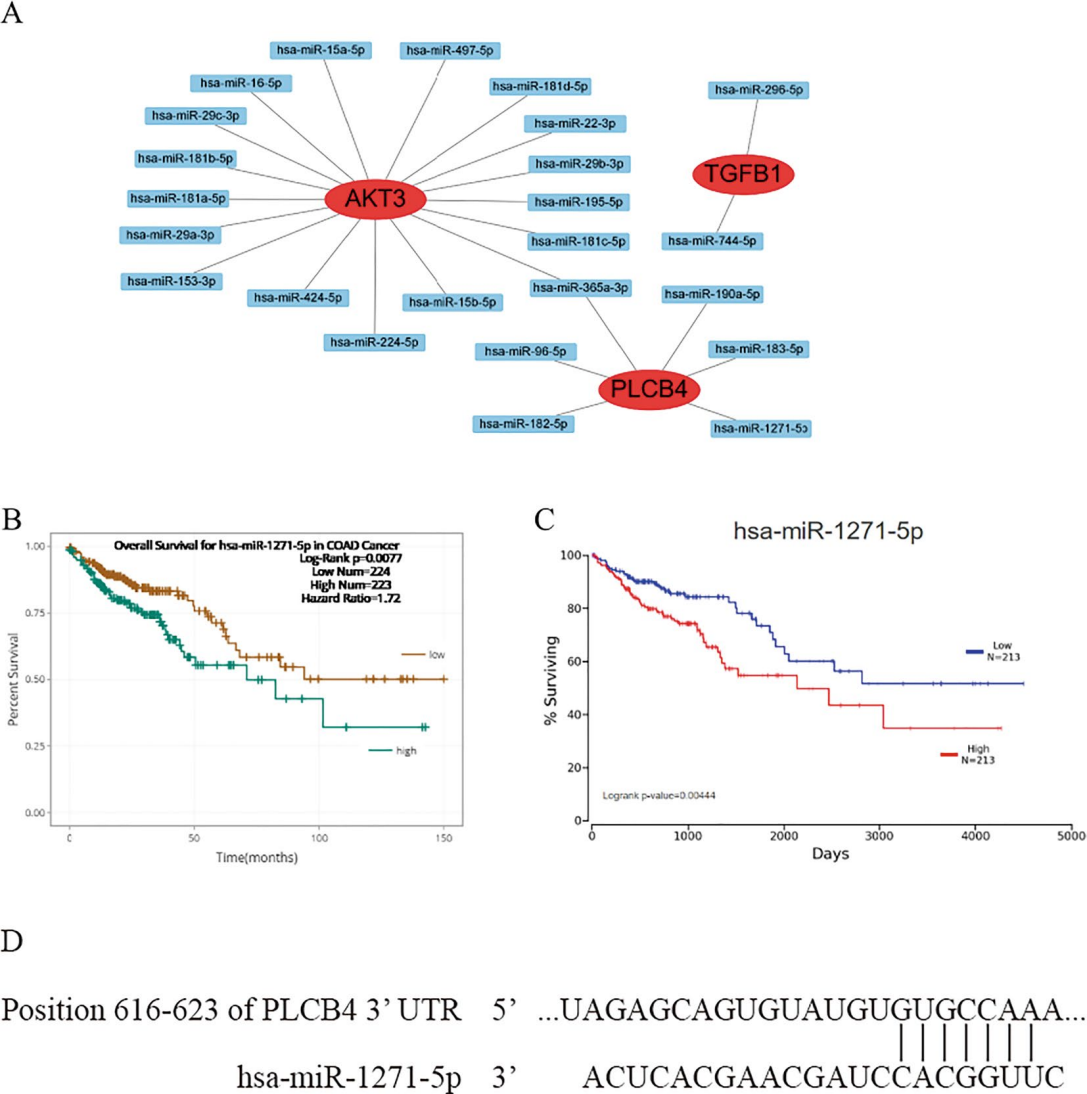
Prediction of miRNA for key genes. (**A**) The miRNA-mRNA interaction network of candidate genes. Significant prognostic values of has-miR-1271-5p on the overall survival of Colon cancer patients by (**B**) ENCORI and (**C**) OncoLnc database. (**D**) The binding sequence between hsa-miR-1271-5p and PLCB4 mRNA.

The ENCORI platform was applied to predict the miRNAs targeting the candidate genes with clinical significance such *as AKT3, TGFB1 and PLCB4* in order to determine possible upstream regulators. We found that 17 miRNAs were possible miRNAs targeting *AKT3*, six miRNAs for *PLCB4*, and two miRNAs for *TGFB1* (Table 4). The miRNA-mRNA interaction network showed that hsa-miR-365a-3p was predicted to target both *AKT3* and *PLCB4* (Fig. 7A).

**Table 4:** Survival analysis of miRNA that targets candidate genes.

Gene name	MicroRNA name	Coef	p value for overall survival (n = 447)
*AKT3*	hsa-miR-15a-5p	− 0.13	0.51000
	hsa-miR-16-5p	− 0.02	0.92000
	hsa-miR-22-3p	0.09	0.64000
	hsa-miR-29a-3p	0.09	0.65000
	hsa-miR-29b-3p	0.13	0.51000
	hsa-miR-181a-5p	0.15	0.45000
	hsa-miR-181b-5p	0.39	0.05400
	hsa-miR-181c-5p	0.30	0.13000
	hsa-miR-224-5p	− 0.19	0.34000
	hsa-miR-15b-5p	− 0.06	0.77000
	hsa-miR-153-3p	0.08	0.71000
	hsa-miR-195-5p	0.11	0.59000
	hsa-miR-29c-3p	0.25	0.22000
	hsa-miR-365a-3p	− 0.15	0.44000
	hsa-miR-424-5p	− 0.05	0.81000
	hsa-miR-497-5p	0.38	0.05700
	hsa-miR-181d-5p	− 0.12	0.53000
*TGFB1*	hsa-miR-296-5p	0.21	0.30000
	hsa-miR-744-5p	− 0.18	0.37000
*PLCB4*	hsa-miR-96-5p	0.15	0.44000
	hsa-miR-182-5p	0.00	0.98000
	hsa-miR-183-5p	− 0.03	0.87000
	hsa-miR-190a-5p	0.17	0.40000
	hsa-miR-365a-3p	− 0.15	0.44000
	hsa-miR-1271-5p	0.54	0.00770*

*p  < 0.05.

### Clinical association of candidate miRNAs

We further validated correlations between OS of colon cancer patients and expression levels of candidate miRNAs by the ENCORI online tool and the OncoLnc database. High hsa-miR-1271-5p expression, which may target *PLCB4*, was significantly associated with poor OS (Fig. 7B,C), but the other 24 miRNAs were not significantly correlated with the OS of colon cancer patients (Table 4). In addition, the binding sites between PLCB4 mRNA and hsa-miR-1217-5p were predicted by the TargetScan website (Fig. 7D).

### KEGG pathway analysis of PLCB4 co-expressed genes and GSEA analysis of PLCB4 in CRC patients based on TCGA data

**Figure 8:**
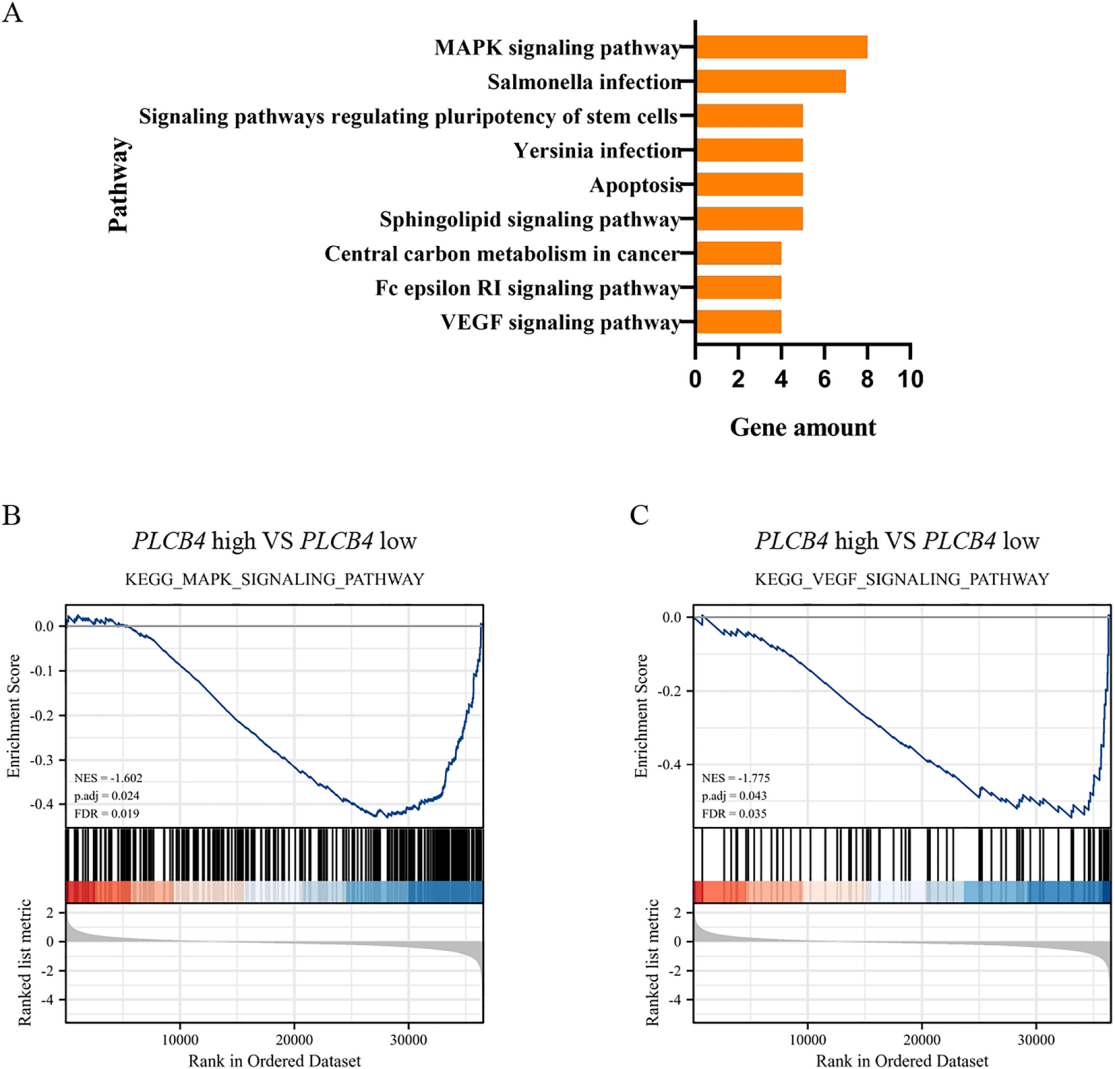
Enrichment analysis of *PLCB4* in colorectal cancer. (**A**) KEGG pathway analysis of top 100 negatively co-expressed genes of *PLCB4* in colorectal cancer identified using the cBioPortal database. GSEA analysis between high (top 50%)—and low (bottom 50%)—*PLCB4* expression using the TCGA COAD data. *PLCB4* low expression group was enriched in the (**B**) MAPK Signaling pathway and (**C**) VEGF signaling pathway.

Co-expression genes of *PLCB4* in CRC was identified using cBioPortal database. The top 100 negatively co-expressed genes were obtained from the Colorectal Adenocarcinoma (TCGA, PanCancer Atlas) cohort (Supplemental Table 3). KEGG pathway enrichment analyses were introduced for a better understanding of these genes through DAVID online tool. There were nine enriched KEGG pathways, including MAPK signaling pathway, salmonella infection, signaling pathways regulating pluripotency of stem cells, Yersinia infection, apoptosis, sphingolipid signaling pathway, central carbon metabolism in cancer, Fc epsilon RI signaling pathway and VEGF signaling pathway, were identified (Fig. 8A). Then, GSEA was applied between the high and low expression levels of *PLCB4* based on TCGA COAD data to verify the KEGG analysis. The results showed that 34 KEGG pathways were closely correlated with *PLCB4* low expression (Supplemental Table 4). We found that MAPK (mitogen‑activated protein kinase) signaling pathway (Fig. 8B) and VEGF (vascular endothelial growth factor) signaling pathway (Fig. 8C) commonly appeared in the enrichment analyses.

## Discussion

OXA-based chemotherapy is one of the most commonly used regimens for mCRC patients^35^. However, OXA resistance develops over time in nearly all patients, leading to treatment failure and resulting in poor prognoses^36^. Many studies have demonstrated some possible mechanisms involved in OXA resistance; for instance, inhibiting *CXCR4/PI3K/AKT1* downstream signaling and blocking the *FOXM1*/*DVL2*/*SNAI1* pathway were elucidated as strategies for overcoming OXA resistance in CRC cells^37,38^. Other study have found that enhancing *Nox1* activation while reducing p38 mitogen-activated protein kinase (MAPK)-dependent escape routes can improve oxaliplatin efficiency^39^. A highly expressed *KLK11* was illustrated to result in chemoresistance via the phosphatidylinositol 3-kinase (PI3K)/Akt signaling pathway^40^. Furthermore, previous research also investigated optimization of the OXA therapeutic effect through combination therapy, including curcumin that modulated the CXC-chemokine/NF-κB signaling pathway^41^, piperlongumine for ROS production^42^, and salidroside to increase apoptosis^43^. Previous research found that biomarkers, including *PKM2*^44^, *SCGB2A1*^45^, *MLKL*, and *CCDC124*^46^, may regulate gene expressions and act as tools to evaluate prognoses or patient responses to OXA treatment. Due to the heterogeneity of cancer cells, OXA resistance has complex mechanisms and individualized effects on each patient. Several molecular mechanisms of OXA resistance were uncovered in CRC, but in-depth research remains to be scrutinized to increase the effectiveness of treatment and prolong survival rates of patients^47^.

The original study of GSE42387 identified resistance-associated genes and pathways by comparing gene and molecular profiles of three different CRC cell lines, including HCT116, HT29, and LoVo, with their respective resistant sub-cell lines. Results demonstrated that no common OXA resistance-related genes were found among the three cell lines. Still, there were intersections in the enrichment analysis, such as response to external stimuli, cytoskeleton, collagen/ECM, metabolism, and ion binding/transport^24^. Notably, HCT116 and HT29 are cell lines derived from primary sites of CRC, while the LoVo cell line is derived from a tumor metastasized to the left supraclavicular lymph node. However, the drug-resistance mechanism of metastatic tumor cells is not yet clear, and remains an issue that urgently needs to be resolved. A more in-depth bioinformatics analysis of the LoVo cell line and its OXA-resistant sub-cell line is needed to better understand the resistance mechanism in mCRC. This study first identified 395 DEGs between parental and resistant cell lines and then constructed a PPI analysis of the DEGs, from which 95 hub genes with high connectivity in the network were screened out, and then we used a KEGG enrichment analysis to reveal the biological functions of the hub genes. In total, 21 key genes were enriched in three pathways. To explore the clinical relevance of these key genes, relationships between survival rates and expression levels of key genes in CRC were analyzed using public databases. Results showed that three candidate genes were significantly related to the prognosis of CRC patients, two of which were upregulated, viz., *AKT3* and *TGFB1*, and one which was downregulated, *PLCB4*. Among them, only *AKT3* was regarded as a significantly deregulated gene in the original study of GSE42387.

The serine/threonine-protein kinase, Akt, also known as protein kinase B (PKB), plays an important role in the PI3K signaling pathway. Many downstream proteins modulated by Akt involve biological functions, such as cellular survival, proliferation, migration, metabolism, and angiogenesis^48,49^. Members of the *AKT* family include three isoforms, *AKT1*, *AKT2*, and *AKT3*, which are involved in different mechanisms of tumor progression^50^. *AKT3* activates several genes associated with the EMT in CRC cells and promotes tumor invasion and metastasis^51^. From an epigenetic point of view, *AKT3* binds to various miRNAs, including miR-125b-5p, miR-424, and miR-384, leading to a decrease in *AKT3* expression, and results in suppression of the growth of CRC cells^52–54^. Some studies showed that long non-coding (lnc)RNAs can bind to miRNAs targeting *AKT3*; as a result, *AKT3* expression was increased and CRC cells proliferation was enhanced^55,56^. In addition, peptidoglycan (PGN), PLK inhibitors (BI 2536, BI 6727, and GSK461364), and propofol (an anesthetic agent) were found to inhibit *AKT3* and may be potential candidates for CRC therapeutic regimens^57–59^.

The *TGFB1*-encoded protein is a cytokine and a member of the transforming growth factor (TGF)-β superfamily. Binding of TGFB1 to TGF-β receptors carries out the TGFB downstream signaling pathway through Smads or DAXX^60,61^. This pathway is involved in many cellular functions, such as cell growth, differentiation, and apoptosis. In the gastrointestinal (GI) tract, TGF-β1 is secreted by various immune cells, stromal cells, and epithelial cells. It acts on all types of gut mucosal cells and is a crucial regulatory factor in maintaining the gut homeostasis that affects inflammation, fibrosis, and cancer. Interestingly, TGF-β1 signaling has a tumor-suppressive effect by inhibiting cell growth and increasing apoptosis in transformed cells during tumor initiation. However, the tumor-suppressive effect is reversed into a tumor-promoting effect in advanced cancer^62^. Recent studies showed that homeobox D9 (*HOXD9*) can increase cell metastasis through the TGF-β1-induced EMT^63^, and the NMYC interactor (NMI) can upregulate signal transducer and activator of transcription 1 (*STAT1*) and then promote tumor cell proliferation via the TGF-β/Smad pathway^64^. However, nitrilase 1 (*NIT1*), which is considered an oncogene, can activate the TGF-β/Smad2/3 pathway and inhibit CRC proliferation^65^. In addition, some studies also revealed epigenetic regulation of the TGF-β pathway by miRNA-500a-5p, which can reduce the EMT mediated by the TGF-β signaling pathway and inhibit invasion by cancer cells^66^. lncRNAs, such as MIR22HG, interact with *SMAD2* to inhibit the TGF-β pathway and reduce the EMT of cancer cells^67^. The circular RNA, CircPACRGL, binds to miR-142-3p and miR-506-3p, which enhances TGF-β1 expression and promotes cancer cell proliferation and metastasis^68^. Some natural substances can inhibit CRC cells by suppressing TGF-β1 signaling, such as ginsenoside Rb2 from ginseng and honokiol (HNK) extracted from magnolia bark, which have the potential to be used to treat CRC patients^69,70^.

*PLCB4* encodes the PLCβ4 protein, one of the isoforms of phospholipase C (PLC). PLCs catalyze PtdIns(4,5)P2 to form two intracellular second messengers, diacylglycerol (DAG) and inositol 1,4,5 trisphosphate (InsP3), which play important roles in signal transduction^71,72^. There are four isomers of PLCs in the PLCβ sub-family, namely PLCβ1, PLCβ2, PLCβ3, and PLCβ4. Although the isomers share some of the conserved structures, each has its own specific cellular and physiological functions^73^. *PLCB1*, *-2*, and *-3* were shown to alter various pathways in different cancers. For instance, *PLCB1* downregulation promotes the Akt/mTOR pathway, which is associated with poor prognoses in patients with myelodysplastic syndrome (MDS)^74,75^. Knockdown of *PLCB2* in melanoma cells activates the Ras/Raf/MAPK pathway, which in turn enhances apoptosis and inhibits cell survival^76^. PLC-beta3-deficient mice develop tumors, such as myeloproliferative disease and lymphomas through the Janus kinase (JAK)/STAT pathway^77^. However, the role of *PLCB4* in CRC and in cancer pathophysiology remain understudied^78^. Our findings suggest that *PLCB4* downregulation is associated with OXA resistance.

Some studies have proposed that epigenetic regulation of non-coding RNA influences the tumorigenesis and resistance of CRC^79^. Increasing evidence have been reported that miRNAs (i.e., miR-34a, miR-143, miR-153, miR-27a, miR-218, and miR-520) play an essential role in tumorigenesis and chemotherapeutic resistance^21^. By identifying these miRs and their targets in OXA resistance/sensitivity, we can determine how they interact with downstream targets, including STAT3, TGF-β, ATG4B, FOXO1, LATS2, NF-κB and so forth^22^. Among the 25 predicted miRNAs in this study, high expression of hsa-miR-1271-5p, which was predicted to target *PLCB4*, was associated with poor OS of colon cancer patients. Many studies investigated the molecular mechanism of miR-1271-5p in tumorigenesis. In ovarian cancer, miR-1271-5p targets *E2F5* which inhibits the mTOR signaling pathway, and as a result, growth of cancer cells was suppressed^80^. It was also suggested that miR-1271-5p binds to *TIAM1* to inactivate the Notch signaling pathway to inhibit the growth and invasion of ovarian cancer cells^81^. In hepatocellular carcinoma, miR-1271-5p increases the sensitivity of cancer cells to radiotherapy by targeting *CDK1*^82^. By targeting *FOXK2*, an oncogene that activates the PI3K/AKT signaling pathway, miR-1271-5p inhibits tumor progression^83^. In addition, miR-1271-5p was shown to exhibit tumor-suppressive functions in studies of acute myeloid leukemia, gliomas, multiple myelomas, lung adenocarcinomas, endometrial carcinoma, and prostate cancer^84–89^. The mechanism of miR-1271-5p in CRC is still obscure. MiR-1271-5p was shown to negatively regulate CD164 expression, and inhibit cancer cell proliferation, migration, invasion, and the EMT^90^. However, in a study using the BET-bromodomain inhibitor, JQ1, in combination with ABT-263 for CRC cells, the synergistic effect of treatment showed that reducing miR-1271-5p increased Noxa protein production and thus promoted the apoptosis of CRC cells^91^. Our findings suggest that high expression of miR-1271-5p, which may target *PLCB4*, leads to increased resistance of mCRC cells to OXA. This linear relationship may play a role in the poor prognosis of colon cancer patients treated with OXA.

To further explore the mechanism of the miR-1271-5p-*PLCB4* axis on OXA resistance in CRC, we conducted KEGG pathway analysis of *PLCB4* co-expressed genes and verified enriched pathways using GSEA analysis of *PLCB4* based on the TCGA database. The results showed that the *PLCB4* low expression was involved in MAPK Signaling pathway and VEGF signaling pathway. MAPK pathway has been demonstrated to contribute to chemotherapy resistance^92^, and recent studies have shown numerous genes to induce OXA resistance via MAPK signaling. The CD44 isoform containing variant exon v6 (CD44v6) plays a critical role in the progression of CRC. Upon cytotoxic stress, CD44v6 contributes to chemoresistance by modulating autophagy, embryonic development, and the PI3K-Akt, MAPK-Ras-Erk pathways^93^. In CRC cells, c-Myb transcription factor prevents oxaliplatin-induced apoptosis, induces reactive oxygen species by boosting NOX1 synthesis, and maintains p38 MAPK as a pro-survival pathway^94^. Forkhead box protein C2 (FOXC2), a member of the forkhead box (Fox) transcription factor family, is reported to induce EMT to promote oxaliplatin resistance by activating the MAPK/ERK signaling pathway^95^. Additionally, studies on Annexin A3 (*ANXA3*) and TOX high mobility group box family member 3 (*TOX3*) have shown that the MAPK signaling pathway is involved in OXA resistance^96,97^. VEGF signal pathway is not only important for tumor angiogenesis, but also for tumor cell survival and growth^98^. Effectors involved in these physiological responses include extracellular signal-regulated kinases (ERKs), Src, phosphoinositide 3-kinase (PI3K)/Akt, focal adhesion kinase (FAK), Rho family GTPases, endothelial NO and p38 mitogen-activated protein kinase (MAPK)^99^. A previous study revealed that OXA exposure induces the expression of VEGF and VEGF receptors (VEGFR) in human colorectal cancer cells, explaining the benefits of anti-VEGF therapy in combination with chemotherapy for CRC patients^100^.

Several limitations should be noted in this study. First, this study included only a single cell line in the analysis, which could solely represent the molecular mechanisms of OXA resistance in patients with supraclavicular lymph node metastasis. In addition, clinical samples from patients with CRC were not obtained for further comparisons. Pathways involved in OXA resistance were not validated by performing molecular-level experiments. However, key genes involved in these pathways were found and may provide candidate mediators for implementing drug discovery. A comprehensive investigation into key genes, associated pathways, and molecular mechanisms in OXA-resistant mCRC should be performed in both the GEO database and clinical samples in future research.

So far, research on *PLCB4* in OXA resistance is limited and may require further studies. Firstly, in vitro functional assays could be helpful in verifying the role of *PLCB4* in OXA resistance. Secondly, it would be better to verify the *PLCB4* as a target gene for hsa-miR-1271-5p in vitro. Finally, clinical samples of CRC from distant lymphatics can be collected to examine relationships between expressions of candidate genes and the efficacy of OXA-based chemotherapy to consolidate the findings.

## Conclusion

In summary, through an integrated bioinformatics analysis, our results suggest that hsa-miR-1271-5p/*PLCB4* is involved in the resistance mechanism of mCRC and may be a therapeutic target to improve the efficacy of OXA.

## Supplementary Information

Supplementary Table 1.

Supplementary Table 2.

Supplementary Table 3.

Supplementary Table 4.

Supplementary Figure 1.

## Data Availability

The dataset supporting the conclusions of this article is included within the article.

## Abbreviations

OXA: Oxaliplatin
mCRC: Metastatic colorectal cancer
GEO: Gene Expression Omnibus
DEGs: Differentially expressed genes
PPIs: Protein–protein interactions
GO: Gene Ontology
KEGG: Kyoto Encyclopedia of Genes and Genomes
GSEA: Gene Set Enrichment Analysis
OS: Overall survival
EMT: Epithelial–mesenchymal transition
miRNAs: MicroRNAs
MFs: Molecular functions
CCs: Cellular component
BPs: Biological process
IC_50_: 50% Inhibitory concentration
FDR: False discovery rate
FC: Fold change
DAVID: The Database for Annotation, Visualization, and Integrated Discovery
KM: Kaplan–Meier
RFS: Relapse-free survival
DFS: Disease-free survival
ENCORI: The Encyclopedia of RNA Interactomes
TCGA: The Cancer Genome Atlas
COAD: Colorectal Adenocarcinoma
