# Supplementary material for: In silico analysis to identify miR-1271-5p/PLCB4 (phospholipase C Beta 4) axis mediated oxaliplatin resistance in metastatic colorectal cancer
Source: Sci Rep. 2023 Mar 16;13:4366. doi: 10.1038/s41598-023-31331-2 (PMC10020571; doi:10.1038/s41598-023-31331-2)
Supplement: Supplementary file 1 — Supplementary Table 1. [file 41598_2023_31331_MOESM1_ESM.docx]

**Supplemental Table 1** List of differentially expressed genes (DEGs).

**Upregulated DEGs**

| **Rank** | **Gene Symbol** | **P.Value** | **logFC** |
| --- | --- | --- | --- |
| 1 | FABP1 | 1.27E-05 | 4.52 |
| 2 | AKR1C1 | 9.16E-05 | 4.00 |
| 3 | NTS | 2.40E-05 | 3.88 |
| 4 | HBE1 | 1.13E-07 | 3.80 |
| 5 | AKR1B10 | 7.32E-05 | 3.68 |
| 6 | AKR1B1 | 5.13E-05 | 3.61 |
| 7 | DHRS2 | 5.27E-06 | 3.49 |
| 8 | AGMO | 2.22E-06 | 3.26 |
| 9 | HBG1 | 1.66E-08 | 3.24 |
| 10 | POTEG | 8.30E-06 | 3.16 |
| 11 | HHLA2 | 1.30E-05 | 2.98 |
| 12 | POTEE | 2.81E-06 | 2.97 |
| 13 | CADPS | 2.34E-07 | 2.91 |
| 14 | ABCC2 | 1.43E-05 | 2.84 |
| 15 | ITGB7 | 5.95E-04 | 2.77 |
| 16 | TUBAL3 | 1.84E-06 | 2.76 |
| 17 | SYTL5 | 1.51E-05 | 2.70 |
| 18 | FST | 2.11E-03 | 2.66 |
| 19 | POTEB | 1.67E-07 | 2.60 |
| 20 | FCGBP | 2.41E-05 | 2.54 |
| 21 | ANKRD30BP2 | 9.12E-05 | 2.48 |
| 22 | RBP1 | 5.55E-06 | 2.39 |
| 23 | CD68 | 4.78E-05 | 2.37 |
| 24 | DQX1 | 3.92E-07 | 2.22 |
| 25 | OTC | 4.24E-05 | 2.19 |
| 26 | C21orf81 | 7.04E-07 | 2.18 |
| 27 | MAGEA2B | 7.48E-07 | 2.08 |
| 28 | PEG10 | 1.28E-05 | 2.02 |
| 29 | SOHLH2 | 6.18E-05 | 2.01 |
| 30 | IFI16 | 4.32E-06 | 1.99 |
| 31 | NPSR1 | 8.84E-06 | 1.98 |
| 32 | POTED | 2.38E-05 | 1.97 |
| 33 | ANXA10 | 2.03E-06 | 1.97 |
| 34 | COL4A5 | 3.63E-06 | 1.92 |
| 35 | ABCB1 | 6.14E-05 | 1.91 |
| 36 | ANKRD20A9P | 1.47E-06 | 1.89 |
| 37 | AKR1C3 | 1.97E-05 | 1.80 |
| 38 | LRIG1 | 2.86E-05 | 1.80 |
| 39 | MYO1A | 2.02E-04 | 1.73 |
| 40 | COL4A6 | 2.97E-05 | 1.67 |
| 41 | KLK9 | 1.19E-03 | 1.65 |
| 42 | HPDL | 2.36E-05 | 1.64 |
| 43 | ST3GAL1 | 4.80E-05 | 1.64 |
| 44 | ASCL2 | 1.03E-02 | 1.63 |
| 45 | C19orf51 | 2.17E-05 | 1.60 |
| 46 | ANG | 3.83E-05 | 1.59 |
| 47 | O3FAR1 | 1.52E-03 | 1.59 |
| 48 | DUSP6 | 9.73E-05 | 1.58 |
| 49 | SRPX | 2.56E-05 | 1.58 |
| 50 | SLC39A5 | 2.03E-05 | 1.57 |
| 51 | FAM198B | 2.21E-04 | 1.55 |
| 52 | INPP5D | 1.07E-03 | 1.54 |
| 53 | PROCR | 3.85E-04 | 1.53 |
| 54 | SPINK4 | 1.34E-03 | 1.53 |
| 55 | ACTL8 | 2.99E-03 | 1.52 |
| 56 | PCK1 | 5.73E-05 | 1.49 |
| 57 | TMEM200A | 1.22E-04 | 1.49 |
| 58 | DAZL | 6.95E-06 | 1.49 |
| 59 | SGK1 | 1.52E-02 | 1.49 |
| 60 | NOXO1 | 2.57E-04 | 1.48 |
| 61 | KIFAP3 | 4.10E-06 | 1.48 |
| 62 | KIAA1199 | 2.63E-04 | 1.48 |
| 63 | C3orf32 | 1.22E-04 | 1.48 |
| 64 | HMGN5 | 5.56E-05 | 1.47 |
| 65 | PTPRR | 4.46E-04 | 1.45 |
| 66 | SAMD3 | 3.26E-04 | 1.44 |
| 67 | UGT1A6 | 2.04E-02 | 1.43 |
| 68 | SOX2 | 8.20E-04 | 1.43 |
| 69 | PLA2G4A | 2.74E-05 | 1.42 |
| 70 | RNASE1 | 3.03E-04 | 1.41 |
| 71 | C21orf90 | 5.65E-04 | 1.40 |
| 72 | DCBLD2 | 3.51E-04 | 1.40 |
| 73 | FTL | 2.01E-04 | 1.38 |
| 74 | CD47 | 6.33E-04 | 1.37 |
| 75 | ST6GALNAC1 | 1.27E-05 | 1.36 |
| 76 | ASB9 | 5.08E-06 | 1.36 |
| 77 | HOXC9 | 4.08E-04 | 1.35 |
| 78 | AKT3 | 6.43E-05 | 1.34 |
| 79 | TNS4 | 2.52E-02 | 1.33 |
| 80 | DAZ2 | 3.74E-06 | 1.33 |
| 81 | HGD | 1.22E-04 | 1.32 |
| 82 | CLRN3 | 7.86E-03 | 1.32 |
| 83 | ALDOC | 4.08E-03 | 1.31 |
| 84 | SLC1A3 | 6.74E-03 | 1.29 |
| 85 | FAM151A | 5.82E-03 | 1.29 |
| 86 | TMPRSS15 | 3.80E-05 | 1.28 |
| 87 | CPE | 8.00E-04 | 1.28 |
| 88 | GALNT5 | 1.13E-02 | 1.27 |
| 89 | PLEKHG5 | 5.72E-03 | 1.26 |
| 90 | KIRREL | 4.15E-04 | 1.26 |
| 91 | RGS2 | 4.41E-02 | 1.25 |
| 92 | TMPRSS4 | 2.87E-05 | 1.24 |
| 93 | CELA3B | 3.24E-04 | 1.23 |
| 94 | DYNLT3 | 1.14E-02 | 1.23 |
| 95 | HYAL1 | 1.88E-05 | 1.23 |
| 96 | NR1I2 | 3.20E-03 | 1.21 |
| 97 | ATL3 | 2.44E-02 | 1.21 |
| 98 | MVP | 6.84E-05 | 1.21 |
| 99 | FLRT3 | 4.88E-03 | 1.20 |
| 100 | SCGB3A2 | 3.21E-03 | 1.20 |
| 101 | FUT3 | 1.55E-04 | 1.19 |
| 102 | PLEC | 8.61E-04 | 1.19 |
| 103 | TNFRSF25 | 5.17E-04 | 1.18 |
| 104 | DHRS9 | 1.73E-05 | 1.18 |
| 105 | C16orf45 | 2.59E-04 | 1.18 |
| 106 | CALB2 | 1.31E-02 | 1.18 |
| 107 | SPANXA1 | 1.11E-03 | 1.17 |
| 108 | CELF2 | 9.96E-03 | 1.17 |
| 109 | DDC | 5.54E-03 | 1.16 |
| 110 | ACE2 | 8.22E-05 | 1.16 |
| 111 | LFNG | 4.53E-03 | 1.15 |
| 112 | SYT1 | 1.95E-05 | 1.15 |
| 113 | HOXC8 | 5.09E-03 | 1.14 |
| 114 | INPP4B | 1.15E-03 | 1.13 |
| 115 | TLCD1 | 3.77E-05 | 1.13 |
| 116 | FXYD4 | 3.73E-05 | 1.13 |
| 117 | POLE2 | 7.50E-03 | 1.12 |
| 118 | GJA1 | 2.25E-05 | 1.12 |
| 119 | SACS | 1.60E-02 | 1.11 |
| 120 | HLA-DQB1 | 9.44E-06 | 1.10 |
| 121 | CDH12 | 1.84E-03 | 1.10 |
| 122 | CPSF6 | 8.33E-04 | 1.10 |
| 123 | POLR3G | 9.50E-03 | 1.09 |
| 124 | TFF3 | 7.96E-05 | 1.09 |
| 125 | SPANXD | 7.04E-05 | 1.09 |
| 126 | RNF217 | 2.70E-05 | 1.07 |
| 127 | RASEF | 2.13E-03 | 1.07 |
| 128 | KRT20 | 3.65E-02 | 1.07 |
| 129 | BTN1A1 | 9.96E-05 | 1.07 |
| 130 | L3MBTL3 | 6.20E-03 | 1.07 |
| 131 | HSPA8 | 4.44E-02 | 1.07 |
| 132 | INSIG1 | 3.43E-02 | 1.06 |
| 133 | SLC39A8 | 1.87E-02 | 1.05 |
| 134 | ARHGAP6 | 1.34E-03 | 1.05 |
| 135 | KLK11 | 1.82E-04 | 1.05 |
| 136 | TGFB1 | 3.52E-04 | 1.04 |
| 137 | MT1L | 1.16E-02 | 1.04 |
| 138 | HOXD13 | 2.58E-04 | 1.04 |
| 139 | EGR1 | 2.82E-03 | 1.04 |
| 140 | SLCO2B1 | 6.27E-05 | 1.04 |
| 141 | PPAP2C | 3.81E-03 | 1.04 |
| 142 | CT45A5 | 1.21E-04 | 1.03 |
| 143 | DDIT4L | 1.54E-04 | 1.02 |
| 144 | NIPAL2 | 8.69E-04 | 1.02 |
| 145 | CMBL | 9.43E-03 | 1.02 |
| 146 | ABCG2 | 3.34E-02 | 1.02 |
| 147 | GPCPD1 | 7.33E-03 | 1.02 |
| 148 | CPOX | 2.78E-02 | 1.02 |
| 149 | PDZD3 | 1.01E-04 | 1.02 |
| 150 | UNC13A | 3.88E-04 | 1.01 |
| 151 | KLK1 | 1.15E-02 | 1.01 |
| 152 | VIL1 | 2.09E-04 | 1.01 |
| 153 | H19 | 5.41E-04 | 1.00 |
| 154 | SERPINB2 | 6.17E-05 | 1.00 |
| 155 | NDUFB6 | 7.03E-03 | 1.00 |

**Downregulated DEGs**

| **Rank** | **Gene Symbol** | **P.Value** | **logFC** |
| --- | --- | --- | --- |
| 1 | INHBA | 7.74E-08 | -5.48 |
| 2 | PCP4 | 6.08E-06 | -4.13 |
| 3 | BEX2 | 2.02E-03 | -4.04 |
| 4 | PRKCDBP | 1.98E-07 | -3.33 |
| 5 | LCN2 | 1.91E-03 | -3.07 |
| 6 | TCEAL8 | 2.88E-07 | -3.01 |
| 7 | ALDH1L2 | 6.23E-04 | -3.00 |
| 8 | ARHGAP23 | 4.00E-04 | -2.93 |
| 9 | KRT80 | 1.31E-03 | -2.90 |
| 10 | CASK | 3.73E-05 | -2.75 |
| 11 | C11orf20 | 7.36E-06 | -2.73 |
| 12 | AMIGO2 | 3.97E-05 | -2.69 |
| 13 | FAM129A | 3.91E-03 | -2.52 |
| 14 | GSTT2 | 2.11E-07 | -2.46 |
| 15 | IGF2 | 1.65E-06 | -2.42 |
| 16 | FSTL1 | 1.70E-07 | -2.40 |
| 17 | SYBU | 8.02E-04 | -2.40 |
| 18 | ALPP | 1.10E-04 | -2.39 |
| 19 | IGFBP1 | 1.03E-04 | -2.36 |
| 20 | KRT34 | 6.54E-04 | -2.36 |
| 21 | CXCR4 | 2.60E-03 | -2.34 |
| 22 | NNMT | 1.32E-02 | -2.33 |
| 23 | ALPPL2 | 1.39E-04 | -2.32 |
| 24 | NUPR1 | 2.34E-02 | -2.31 |
| 25 | C4orf7 | 6.03E-05 | -2.30 |
| 26 | LHFP | 6.78E-06 | -2.29 |
| 27 | TFF2 | 9.78E-05 | -2.27 |
| 28 | ARRDC4 | 8.49E-04 | -2.22 |
| 29 | TFF1 | 1.18E-05 | -2.19 |
| 30 | TXNIP | 3.18E-02 | -2.16 |
| 31 | ECHDC2 | 1.49E-06 | -2.12 |
| 32 | LGALS2 | 1.83E-04 | -2.11 |
| 33 | IFIT1 | 1.13E-03 | -2.11 |
| 34 | PI3 | 1.34E-02 | -2.06 |
| 35 | CCDC85A | 3.26E-03 | -2.05 |
| 36 | HSPB3 | 1.46E-06 | -2.04 |
| 37 | GNG11 | 8.97E-05 | -2.03 |
| 38 | VIM | 3.70E-05 | -2.02 |
| 39 | GUCY1A3 | 4.82E-07 | -2.01 |
| 40 | FMN1 | 1.45E-06 | -1.97 |
| 41 | MEIS3P1 | 9.58E-06 | -1.94 |
| 42 | GPR56 | 8.42E-04 | -1.92 |
| 43 | STAG2 | 1.33E-04 | -1.92 |
| 44 | RTP4 | 1.70E-04 | -1.92 |
| 45 | SCARA3 | 1.41E-05 | -1.92 |
| 46 | UCHL1 | 9.01E-07 | -1.91 |
| 47 | PRSS23 | 4.56E-05 | -1.90 |
| 48 | CIB2 | 1.64E-03 | -1.88 |
| 49 | MFAP5 | 7.02E-05 | -1.85 |
| 50 | C16orf48 | 4.50E-05 | -1.83 |
| 51 | DLEC1 | 1.75E-04 | -1.83 |
| 52 | SLPI | 4.90E-03 | -1.80 |
| 53 | FOLR1 | 3.29E-06 | -1.79 |
| 54 | DIO3OS | 2.45E-06 | -1.79 |
| 55 | CD200 | 2.24E-05 | -1.77 |
| 56 | DIO3 | 8.87E-07 | -1.75 |
| 57 | DOK7 | 1.61E-02 | -1.75 |
| 58 | SPINK1 | 1.04E-02 | -1.75 |
| 59 | EMB | 2.81E-06 | -1.74 |
| 60 | UNC5B | 7.47E-03 | -1.74 |
| 61 | DTX3 | 2.95E-03 | -1.69 |
| 62 | CD36 | 3.69E-06 | -1.66 |
| 63 | EDN1 | 5.35E-03 | -1.66 |
| 64 | IGFBP2 | 5.47E-04 | -1.65 |
| 65 | SOD3 | 4.97E-05 | -1.64 |
| 66 | CYP2J2 | 1.65E-02 | -1.64 |
| 67 | GPRIN2 | 4.85E-04 | -1.62 |
| 68 | SYNE1 | 1.99E-04 | -1.62 |
| 69 | LHX2 | 6.92E-04 | -1.57 |
| 70 | DEF6 | 5.23E-04 | -1.57 |
| 71 | IL20RB | 2.01E-02 | -1.57 |
| 72 | CRABP1 | 9.18E-04 | -1.56 |
| 73 | BDKRB2 | 1.16E-04 | -1.56 |
| 74 | KRT4 | 1.15E-03 | -1.56 |
| 75 | KRCC1 | 5.73E-04 | -1.56 |
| 76 | PID1 | 6.85E-06 | -1.55 |
| 77 | GAB2 | 4.63E-03 | -1.55 |
| 78 | ODAM | 1.81E-03 | -1.52 |
| 79 | BICC1 | 4.92E-05 | -1.52 |
| 80 | DAPK1 | 2.03E-03 | -1.52 |
| 81 | FOXP2 | 2.94E-04 | -1.52 |
| 82 | SH3BGRL | 8.36E-04 | -1.51 |
| 83 | SNTB1 | 1.55E-02 | -1.51 |
| 84 | SLC43A1 | 7.09E-03 | -1.51 |
| 85 | DHX58 | 3.65E-03 | -1.50 |
| 86 | FAM113B | 4.03E-03 | -1.49 |
| 87 | PPFIBP1 | 2.26E-02 | -1.49 |
| 88 | GLIS3 | 1.19E-03 | -1.49 |
| 89 | GFOD1 | 1.81E-03 | -1.49 |
| 90 | RARRES3 | 1.52E-03 | -1.49 |
| 91 | RBKS | 3.14E-03 | -1.47 |
| 92 | LGR6 | 2.73E-05 | -1.47 |
| 93 | EMX2OS | 2.51E-03 | -1.46 |
| 94 | EPS8L1 | 3.90E-03 | -1.46 |
| 95 | APBB2 | 1.23E-03 | -1.45 |
| 96 | TNNC1 | 7.01E-05 | -1.45 |
| 97 | S100A13 | 3.11E-05 | -1.45 |
| 98 | FANCF | 1.17E-03 | -1.44 |
| 99 | IFIT2 | 5.00E-05 | -1.43 |
| 100 | FLNB | 8.63E-03 | -1.42 |
| 101 | APOBEC3C | 2.58E-03 | -1.42 |
| 102 | IL18 | 1.74E-03 | -1.42 |
| 103 | CARD14 | 7.58E-03 | -1.42 |
| 104 | RGS11 | 5.11E-03 | -1.41 |
| 105 | C1orf186 | 4.79E-05 | -1.41 |
| 106 | SLC38A5 | 1.29E-04 | -1.41 |
| 107 | APOBEC3F | 4.71E-05 | -1.41 |
| 108 | ACOT4 | 3.11E-06 | -1.40 |
| 109 | SP5 | 7.15E-04 | -1.40 |
| 110 | FAM59A | 6.12E-03 | -1.40 |
| 111 | TGFBI | 1.14E-05 | -1.40 |
| 112 | TMSB15B | 5.27E-04 | -1.40 |
| 113 | THBS1 | 4.03E-05 | -1.39 |
| 114 | XAGE2B | 1.35E-03 | -1.38 |
| 115 | NRXN3 | 3.72E-05 | -1.38 |
| 116 | NHS | 6.09E-06 | -1.37 |
| 117 | C21orf88 | 2.91E-03 | -1.37 |
| 118 | BEST1 | 1.70E-02 | -1.37 |
| 119 | MBNL2 | 1.87E-04 | -1.36 |
| 120 | TLR2 | 2.27E-03 | -1.36 |
| 121 | FRAS1 | 2.18E-04 | -1.35 |
| 122 | FYN | 5.05E-04 | -1.35 |
| 123 | C10orf54 | 5.73E-04 | -1.35 |
| 124 | SSPN | 1.92E-04 | -1.34 |
| 125 | ZNF185 | 6.79E-05 | -1.34 |
| 126 | DNAH14 | 1.38E-04 | -1.34 |
| 127 | BTBD16 | 8.03E-05 | -1.34 |
| 128 | ZNF226 | 5.04E-05 | -1.33 |
| 129 | LAMP3 | 4.93E-03 | -1.33 |
| 130 | UST | 2.66E-03 | -1.33 |
| 131 | SELS | 8.22E-03 | -1.32 |
| 132 | SLC15A3 | 7.24E-04 | -1.31 |
| 133 | XAF1 | 3.72E-04 | -1.30 |
| 134 | PDZK1IP1 | 2.50E-04 | -1.30 |
| 135 | LGALS8 | 1.36E-04 | -1.30 |
| 136 | CRIP1 | 3.48E-05 | -1.29 |
| 137 | FAM84A | 3.67E-05 | -1.29 |
| 138 | ZCWPW1 | 5.47E-03 | -1.29 |
| 139 | CYP3A5 | 2.40E-03 | -1.29 |
| 140 | TREX1 | 2.16E-04 | -1.28 |
| 141 | ISG20 | 1.35E-04 | -1.28 |
| 142 | C9orf150 | 4.78E-02 | -1.28 |
| 143 | THSD4 | 3.19E-04 | -1.28 |
| 144 | EHD2 | 3.34E-05 | -1.27 |
| 145 | ARHGAP29 | 5.36E-04 | -1.26 |
| 146 | FGF18 | 1.41E-04 | -1.26 |
| 147 | PALLD | 9.36E-06 | -1.25 |
| 148 | ABLIM2 | 5.11E-05 | -1.25 |
| 149 | FAM189A1 | 1.71E-05 | -1.25 |
| 150 | FAM134B | 2.16E-02 | -1.24 |
| 151 | MORC4 | 9.46E-03 | -1.24 |
| 152 | PLCD1 | 1.04E-03 | -1.23 |
| 153 | OGDHL | 1.56E-03 | -1.22 |
| 154 | SYTL1 | 1.58E-02 | -1.22 |
| 155 | NR2E3 | 5.60E-05 | -1.22 |
| 156 | CRLF1 | 1.32E-03 | -1.21 |
| 157 | TNFRSF14 | 3.24E-05 | -1.21 |
| 158 | GAMT | 1.72E-03 | -1.21 |
| 159 | CHADL | 6.59E-05 | -1.21 |
| 160 | PTPRO | 2.48E-05 | -1.20 |
| 161 | TMPRSS3 | 3.32E-04 | -1.19 |
| 162 | BACE1 | 2.40E-03 | -1.19 |
| 163 | PLCB4 | 2.17E-03 | -1.19 |
| 164 | SLCO2A1 | 1.47E-03 | -1.19 |
| 165 | PRTFDC1 | 2.76E-03 | -1.19 |
| 166 | MAOA | 1.36E-02 | -1.18 |
| 167 | EMP1 | 3.68E-03 | -1.18 |
| 168 | TNAP | 9.61E-06 | -1.17 |
| 169 | TUBB2B | 4.50E-03 | -1.17 |
| 170 | RPS6KA5 | 1.06E-02 | -1.17 |
| 171 | TOM1L2 | 1.29E-02 | -1.17 |
| 172 | MAGED1 | 9.68E-04 | -1.16 |
| 173 | SLC24A1 | 2.91E-02 | -1.16 |
| 174 | CCL26 | 3.25E-02 | -1.16 |
| 175 | CEBPD | 3.08E-02 | -1.16 |
| 176 | CDK14 | 1.26E-04 | -1.16 |
| 177 | SEMG1 | 4.49E-03 | -1.16 |
| 178 | CTDSPL | 1.46E-03 | -1.15 |
| 179 | PSKH1 | 1.89E-02 | -1.15 |
| 180 | EMBP1 | 2.02E-03 | -1.15 |
| 181 | FOSL2 | 7.85E-03 | -1.15 |
| 182 | MGLL | 2.84E-03 | -1.15 |
| 183 | TMEM133 | 2.77E-03 | -1.14 |
| 184 | CYP3A7 | 8.51E-04 | -1.13 |
| 185 | RPS6KA2 | 7.14E-03 | -1.13 |
| 186 | IFITM3 | 9.40E-04 | -1.13 |
| 187 | CLDN2 | 3.30E-03 | -1.12 |
| 188 | MYLK | 4.62E-04 | -1.12 |
| 189 | IFITM2 | 6.82E-04 | -1.12 |
| 190 | CCDC80 | 1.56E-04 | -1.11 |
| 191 | LAMA3 | 6.19E-03 | -1.11 |
| 192 | CCNYL1 | 2.95E-05 | -1.11 |
| 193 | IFITM4P | 8.95E-04 | -1.10 |
| 194 | SPON1 | 1.31E-05 | -1.10 |
| 195 | SEMA3B | 5.78E-04 | -1.09 |
| 196 | HIST1H4F | 7.43E-03 | -1.09 |
| 197 | KCNJ8 | 1.33E-05 | -1.09 |
| 198 | TMEM173 | 7.14E-03 | -1.09 |
| 199 | TMEM61 | 4.52E-04 | -1.09 |
| 200 | GEM | 2.29E-03 | -1.09 |
| 201 | ARHGEF37 | 1.30E-02 | -1.09 |
| 202 | SRGAP1 | 4.33E-02 | -1.09 |
| 203 | TRIM45 | 4.10E-02 | -1.08 |
| 204 | SLC25A5 | 8.30E-03 | -1.08 |
| 205 | CDH11 | 7.10E-05 | -1.07 |
| 206 | RASIP1 | 1.31E-02 | -1.07 |
| 207 | ANO1 | 3.22E-04 | -1.06 |
| 208 | SLFN5 | 8.97E-03 | -1.06 |
| 209 | FAAH2 | 4.72E-03 | -1.05 |
| 210 | RUNX1 | 1.28E-03 | -1.05 |
| 211 | PF4 | 1.25E-04 | -1.05 |
| 212 | EPHA10 | 5.66E-03 | -1.05 |
| 213 | CLDN3 | 1.49E-03 | -1.05 |
| 214 | BDKRB1 | 8.74E-04 | -1.04 |
| 215 | IRS1 | 1.21E-04 | -1.04 |
| 216 | FGF2 | 2.33E-02 | -1.04 |
| 217 | OBSL1 | 8.28E-03 | -1.04 |
| 218 | LRRC56 | 1.47E-02 | -1.04 |
| 219 | PTPN13 | 1.65E-03 | -1.03 |
| 220 | GTPBP2 | 3.82E-02 | -1.03 |
| 221 | ARHGEF17 | 9.45E-05 | -1.03 |
| 222 | IFITM1 | 6.62E-03 | -1.03 |
| 223 | DNAJB9 | 5.15E-03 | -1.03 |
| 224 | ADRA2C | 4.27E-03 | -1.03 |
| 225 | DDX3Y | 3.18E-04 | -1.03 |
| 226 | C21orf67 | 3.74E-02 | -1.03 |
| 227 | TPM2 | 7.95E-04 | -1.03 |
| 228 | MFSD6L | 7.63E-04 | -1.03 |
| 229 | FLJ45340 | 7.37E-03 | -1.03 |
| 230 | NOXA1 | 1.43E-02 | -1.02 |
| 231 | RFX3 | 5.96E-03 | -1.02 |
| 232 | TRIM68 | 5.19E-04 | -1.02 |
| 233 | BTLA | 1.26E-05 | -1.02 |
| 234 | HSD17B2 | 3.23E-03 | -1.02 |
| 235 | SORL1 | 8.39E-03 | -1.01 |
| 236 | MERTK | 1.14E-02 | -1.01 |
| 237 | C9orf103 | 2.97E-02 | -1.01 |
| 238 | PTPRH | 1.73E-03 | -1.00 |
| 239 | ATP6V1E2 | 3.45E-04 | -1.00 |
| 240 | PDE4B | 4.52E-05 | -1.00 |
