# Supplementary material for: In silico analysis to identify miR-1271-5p/PLCB4 (phospholipase C Beta 4) axis mediated oxaliplatin resistance in metastatic colorectal cancer
Source: Sci Rep. 2023 Mar 16;13:4366. doi: 10.1038/s41598-023-31331-2 (PMC10020571; doi:10.1038/s41598-023-31331-2)
Supplement: Supplementary file 2 — Supplementary Table 2. [file 41598_2023_31331_MOESM2_ESM.docx]

**Supplemental Table 2** Enriched Gene-Ontologies (GO’s) for 95 hub genes.

| **Category** | **Term** | **Count** | **PValue** | **FDR** |
| --- | --- | --- | --- | --- |
| BP | GO:0060337~type I interferon signaling pathway | 8 | 6.11E-08 | 6.18E-05 |
| BP | GO:0009615~response to virus | 9 | 1.68E-07 | 8.51E-05 |
| CC | GO:0005615~extracellular space | 22 | 3.84E-06 | 6.37E-04 |
| BP | GO:0044597~daunorubicin metabolic process | 4 | 9.03E-06 | 2.28E-03 |
| BP | GO:0044598~doxorubicin metabolic process | 4 | 9.03E-06 | 2.28E-03 |
| BP | GO:0035456~response to interferon-beta | 4 | 1.35E-05 | 2.73E-03 |
| CC | GO:0070062~extracellular exosome | 32 | 1.73E-05 | 1.44E-03 |
| BP | GO:0051607~defense response to virus | 8 | 3.63E-05 | 6.12E-03 |
| BP | GO:0045071~negative regulation of viral genome replication | 5 | 6.92E-05 | 1.00E-02 |
| MF | GO:0004033~aldo-keto reductase (NADP) activity | 4 | 7.02E-05 | 1.28E-02 |
| CC | GO:0005886~plasma membrane | 39 | 8.05E-05 | 3.94E-03 |
| MF | GO:0047718~indanol dehydrogenase activity | 3 | 8.98E-05 | 1.28E-02 |
| BP | GO:0008202~steroid metabolic process | 5 | 9.22E-05 | 1.15E-02 |
| CC | GO:0045121~membrane raft | 8 | 9.49E-05 | 3.94E-03 |
| BP | GO:0051897~positive regulation of protein kinase B signaling | 6 | 1.02E-04 | 1.15E-02 |
| BP | GO:0007269~neurotransmitter secretion | 5 | 1.81E-04 | 1.83E-02 |
| CC | GO:0005882~intermediate filament | 6 | 2.97E-04 | 9.85E-03 |
| BP | GO:0048661~positive regulation of smooth muscle cell proliferation | 5 | 3.39E-04 | 3.12E-02 |
| BP | GO:0007586~digestion | 5 | 4.09E-04 | 3.45E-02 |
| MF | GO:0004032~alditol:NADP+ 1-oxidoreductase activity | 3 | 6.19E-04 | 4.41E-02 |
| MF | GO:0047035~testosterone dehydrogenase (NAD+) activity | 3 | 6.19E-04 | 4.41E-02 |
| BP | GO:0042908~xenobiotic transport | 3 | 6.26E-04 | 4.87E-02 |
| BP | GO:0007204~positive regulation of cytosolic calcium ion concentration | 6 | 8.90E-04 | 6.20E-02 |
| BP | GO:0006805~xenobiotic metabolic process | 5 | 9.20E-04 | 6.20E-02 |
| BP | GO:0042448~progesterone metabolic process | 3 | 1.07E-03 | 6.74E-02 |
| BP | GO:0006954~inflammatory response | 9 | 1.20E-03 | 7.14E-02 |
| BP | GO:0035455~response to interferon-alpha | 3 | 1.33E-03 | 7.46E-02 |
| BP | GO:0061045~negative regulation of wound healing | 3 | 1.62E-03 | 8.61E-02 |
| BP | GO:0002576~platelet degranulation | 5 | 2.57E-03 | 1.22E-01 |
| BP | GO:0009607~response to biotic stimulus | 3 | 2.64E-03 | 1.22E-01 |
| BP | GO:0043537~negative regulation of blood vessel endothelial cell migration | 3 | 2.64E-03 | 1.22E-01 |
| CC | GO:0005578~proteinaceous extracellular matrix | 7 | 2.64E-03 | 6.81E-02 |
| CC | GO:0031093~platelet alpha granule lumen | 4 | 2.87E-03 | 6.81E-02 |
| MF | GO:0030169~low-density lipoprotein particle binding | 3 | 3.01E-03 | 1.72E-01 |
| BP | GO:0010628~positive regulation of gene expression | 7 | 3.35E-03 | 1.46E-01 |
| BP | GO:0010744~positive regulation of macrophage derived foam cell differentiation | 3 | 3.46E-03 | 1.46E-01 |
| BP | GO:0071407~cellular response to organic cyclic compound | 4 | 4.27E-03 | 1.64E-01 |
| BP | GO:0008284~positive regulation of cell proliferation | 9 | 4.30E-03 | 1.64E-01 |
| CC | GO:0031012~extracellular matrix | 7 | 4.30E-03 | 8.93E-02 |
| BP | GO:0046597~negative regulation of viral entry into host cell | 3 | 4.38E-03 | 1.64E-01 |
| BP | GO:0043536~positive regulation of blood vessel endothelial cell migration | 3 | 4.88E-03 | 1.70E-01 |
| BP | GO:0006855~drug transmembrane transport | 3 | 4.88E-03 | 1.70E-01 |
| BP | GO:0019229~regulation of vasoconstriction | 3 | 5.40E-03 | 1.82E-01 |
| MF | GO:0001948~glycoprotein binding | 4 | 5.52E-03 | 2.26E-01 |
| MF | GO:0005515~protein binding | 62 | 5.56E-03 | 2.26E-01 |
| BP | GO:0009749~response to glucose | 4 | 6.36E-03 | 2.07E-01 |
| CC | GO:0009986~cell surface | 9 | 6.91E-03 | 1.27E-01 |
| BP | GO:0034341~response to interferon-gamma | 3 | 7.74E-03 | 2.45E-01 |
