# Supplementary material for: In silico analysis to identify miR-1271-5p/PLCB4 (phospholipase C Beta 4) axis mediated oxaliplatin resistance in metastatic colorectal cancer
Source: Sci Rep. 2023 Mar 16;13:4366. doi: 10.1038/s41598-023-31331-2 (PMC10020571; doi:10.1038/s41598-023-31331-2)
Supplement: Supplementary file 3 — Supplementary Table 3. [file 41598_2023_31331_MOESM3_ESM.docx]

**Supplemental Table 3** The Top 100 negative co-expressed genes of PLCB4.

| **NO.** | **Correlated Gene** | **Spearman's Correlation** | **p-Value** | **q-Value** |
| --- | --- | --- | --- | --- |
| 1 | GAS2L1 | -0.51204 | 2.32E-36 | 2.31E-33 |
| 2 | MAP3K6 | -0.49149 | 3.22E-33 | 1.88E-30 |
| 3 | TTC7A | -0.48453 | 3.34E-32 | 1.55E-29 |
| 4 | NRGN | -0.48024 | 1.38E-31 | 5.96E-29 |
| 5 | CDR2L | -0.47867 | 2.31E-31 | 9.57E-29 |
| 6 | TRIM7 | -0.4723 | 1.80E-30 | 7.03E-28 |
| 7 | SZRD1 | -0.47016 | 3.57E-30 | 1.29E-27 |
| 8 | CTXN1 | -0.46512 | 1.74E-29 | 5.40E-27 |
| 9 | METRN | -0.45857 | 1.31E-28 | 3.67E-26 |
| 10 | PKM | -0.45653 | 2.44E-28 | 6.74E-26 |
| 11 | HAGHL | -0.44798 | 3.14E-27 | 6.94E-25 |
| 12 | MT2A | -0.4478 | 3.31E-27 | 7.17E-25 |
| 13 | CDC42EP1 | -0.44504 | 7.44E-27 | 1.48E-24 |
| 14 | SLC66A2 | -0.44407 | 9.88E-27 | 1.94E-24 |
| 15 | TEDC1 | -0.4348 | 1.41E-25 | 2.24E-23 |
| 16 | TFAP2A | -0.43423 | 1.65E-25 | 2.58E-23 |
| 17 | TRNP1 | -0.43049 | 4.71E-25 | 6.94E-23 |
| 18 | PLLP | -0.42886 | 7.41E-25 | 1.05E-22 |
| 19 | ZFYVE19 | -0.42715 | 1.19E-24 | 1.65E-22 |
| 20 | S100A16 | -0.42679 | 1.31E-24 | 1.80E-22 |
| 21 | CAMK2N2 | -0.42572 | 1.75E-24 | 2.32E-22 |
| 22 | PFKP | -0.42556 | 1.83E-24 | 2.41E-22 |
| 23 | MYRF | -0.42485 | 2.23E-24 | 2.89E-22 |
| 24 | KCTD1 | -0.42265 | 4.05E-24 | 5.06E-22 |
| 25 | LMF2 | -0.42023 | 7.78E-24 | 9.49E-22 |
| 26 | ISG20 | -0.41625 | 2.25E-23 | 2.59E-21 |
| 27 | MAPK12 | -0.416 | 2.40E-23 | 2.73E-21 |
| 28 | ANXA10 | -0.41541 | 3.69E-16 | 1.25E-14 |
| 29 | LPCAT1 | -0.41271 | 5.72E-23 | 6.02E-21 |
| 30 | TRPV6 | -0.41267 | 5.79E-23 | 6.05E-21 |
| 31 | SNAI3 | -0.41024 | 1.09E-22 | 1.12E-20 |
| 32 | FUOM | -0.40811 | 1.89E-22 | 1.90E-20 |
| 33 | MAPK11 | -0.40778 | 2.06E-22 | 2.05E-20 |
| 34 | VPS18 | -0.40442 | 4.88E-22 | 4.66E-20 |
| 35 | CRIP1 | -0.40387 | 5.60E-22 | 5.28E-20 |
| 36 | EHD1 | -0.40356 | 6.07E-22 | 5.61E-20 |
| 37 | GAD1 | -0.4015 | 1.02E-21 | 9.18E-20 |
| 38 | RELT | -0.40133 | 1.07E-21 | 9.52E-20 |
| 39 | CCK | -0.40082 | 4.69E-15 | 1.27E-13 |
| 40 | RHOF | -0.40078 | 1.22E-21 | 1.09E-19 |
| 41 | RAMP1 | -0.40043 | 1.34E-21 | 1.17E-19 |
| 42 | ARHGAP10 | -0.39644 | 3.62E-21 | 3.01E-19 |
| 43 | ZDHHC18 | -0.39503 | 5.14E-21 | 4.20E-19 |
| 44 | DNAAF3 | -0.39326 | 7.93E-21 | 6.38E-19 |
| 45 | TNNT1 | -0.39294 | 8.60E-21 | 6.76E-19 |
| 46 | ALOXE3 | -0.39291 | 8.65E-21 | 6.77E-19 |
| 47 | SLC25A22 | -0.39156 | 1.20E-20 | 9.21E-19 |
| 48 | NFKB2 | -0.39099 | 1.38E-20 | 1.05E-18 |
| 49 | IFI30 | -0.38985 | 1.83E-20 | 1.33E-18 |
| 50 | RAC3 | -0.38884 | 2.33E-20 | 1.67E-18 |
| 51 | OSR2 | -0.38878 | 2.36E-20 | 1.69E-18 |
| 52 | FSCN1 | -0.38749 | 3.23E-20 | 2.23E-18 |
| 53 | PKN3 | -0.38713 | 3.51E-20 | 2.41E-18 |
| 54 | APC2 | -0.38709 | 3.55E-20 | 2.43E-18 |
| 55 | ZMYND15 | -0.38683 | 3.78E-20 | 2.58E-18 |
| 56 | ATP8B3 | -0.38662 | 3.97E-20 | 2.68E-18 |
| 57 | LMNA | -0.38623 | 4.37E-20 | 2.93E-18 |
| 58 | KLC2 | -0.3859 | 4.72E-20 | 3.13E-18 |
| 59 | UROD | -0.3851 | 5.71E-20 | 3.77E-18 |
| 60 | RHBDF2 | -0.38496 | 5.91E-20 | 3.88E-18 |
| 61 | CHRFAM7A | -0.3846 | 6.45E-20 | 4.18E-18 |
| 62 | PARP12 | -0.38293 | 9.58E-20 | 6.01E-18 |
| 63 | GMPPA | -0.38153 | 1.33E-19 | 8.28E-18 |
| 64 | RASSF1 | -0.38113 | 1.46E-19 | 9.04E-18 |
| 65 | ACP2 | -0.38022 | 1.82E-19 | 1.11E-17 |
| 66 | MT1X | -0.37981 | 2.00E-19 | 1.20E-17 |
| 67 | CHST6 | -0.3791 | 2.36E-19 | 1.40E-17 |
| 68 | PRF1 | -0.37845 | 2.75E-19 | 1.61E-17 |
| 69 | PPCDC | -0.3782 | 2.91E-19 | 1.69E-17 |
| 70 | PLXND1 | -0.37794 | 3.09E-19 | 1.79E-17 |
| 71 | CYB5R3 | -0.3777 | 3.26E-19 | 1.88E-17 |
| 72 | DUSP4 | -0.37728 | 3.61E-19 | 2.06E-17 |
| 73 | LINC01547 | -0.37701 | 3.83E-19 | 2.17E-17 |
| 74 | RNF125 | -0.3758 | 5.07E-19 | 2.84E-17 |
| 75 | WDR54 | -0.37557 | 5.35E-19 | 2.98E-17 |
| 76 | COTL1 | -0.3747 | 6.55E-19 | 3.54E-17 |
| 77 | RNF19B | -0.3743 | 7.17E-19 | 3.84E-17 |
| 78 | PLOD1 | -0.37345 | 8.71E-19 | 4.57E-17 |
| 79 | SEMA4B | -0.37237 | 1.12E-18 | 5.74E-17 |
| 80 | AGAP2-AS1 | -0.37111 | 1.49E-18 | 7.38E-17 |
| 81 | GJB5 | -0.37026 | 1.80E-18 | 8.89E-17 |
| 82 | DUSP7 | -0.37004 | 1.90E-18 | 9.30E-17 |
| 83 | PGAM1 | -0.36904 | 2.38E-18 | 1.14E-16 |
| 84 | TUBA1B | -0.36842 | 2.73E-18 | 1.30E-16 |
| 85 | TYMS | -0.3684 | 2.74E-18 | 1.30E-16 |
| 86 | RHOG | -0.36825 | 2.84E-18 | 1.34E-16 |
| 87 | CENPM | -0.36668 | 4.04E-18 | 1.86E-16 |
| 88 | RAB3A | -0.36648 | 4.22E-18 | 1.94E-16 |
| 89 | MGAT3 | -0.36578 | 4.94E-18 | 2.26E-16 |
| 90 | AKT1 | -0.36499 | 5.88E-18 | 2.68E-16 |
| 91 | MTCL1 | -0.36424 | 6.95E-18 | 3.16E-16 |
| 92 | OTX1 | -0.36415 | 7.09E-18 | 3.20E-16 |
| 93 | GPR3 | -0.36399 | 7.35E-18 | 3.30E-16 |
| 94 | FLVCR2 | -0.36365 | 7.93E-18 | 3.55E-16 |
| 95 | PAK6 | -0.36344 | 8.31E-18 | 3.70E-16 |
| 96 | EMC9 | -0.36256 | 1.01E-17 | 4.47E-16 |
| 97 | BAG2 | -0.36212 | 1.11E-17 | 4.91E-16 |
| 98 | PNMA1 | -0.36147 | 1.28E-17 | 5.61E-16 |
| 99 | CD82 | -0.36084 | 1.47E-17 | 6.44E-16 |
| 100 | CTSD | -0.36022 | 1.69E-17 | 7.30E-16 |
