# Supplementary material for: In silico analysis to identify miR-1271-5p/PLCB4 (phospholipase C Beta 4) axis mediated oxaliplatin resistance in metastatic colorectal cancer
Source: Sci Rep. 2023 Mar 16;13:4366. doi: 10.1038/s41598-023-31331-2 (PMC10020571; doi:10.1038/s41598-023-31331-2)
Supplement: Supplementary file 4 — Supplementary Table 4. [file 41598_2023_31331_MOESM4_ESM.docx]

**Supplemental Table 4** GSEA analysis grouped by PLCB4 expression level in CRC patients.

| **KEGG name** | **NES** | ***P* adjust** | ***q*-values** |
| --- | --- | --- | --- |
| MAPK_SIGNALING_PATHWAY | -1.60 | 0.0236 | 0.0192 |
| NEUROACTIVE_LIGAND_RECEPTOR_INTERACTION | -1.60 | 0.0236 | 0.0192 |
| CYTOKINE_CYTOKINE_RECEPTOR_INTERACTION | -2.08 | 0.0236 | 0.0192 |
| FOCAL_ADHESION | -1.59 | 0.0236 | 0.0192 |
| CHEMOKINE_SIGNALING_PATHWAY | -2.04 | 0.0236 | 0.0192 |
| CALCIUM_SIGNALING_PATHWAY | -1.63 | 0.0236 | 0.0192 |
| SYSTEMIC_LUPUS_ERYTHEMATOSUS | -1.97 | 0.0236 | 0.0192 |
| NATURAL_KILLER_CELL_MEDIATED_CYTOTOXICITY | -2.26 | 0.0236 | 0.0192 |
| CELL_ADHESION_MOLECULES_CAMS | -2.06 | 0.0236 | 0.0192 |
| MELANOGENESIS | -1.69 | 0.0236 | 0.0192 |
| TOLL_LIKE_RECEPTOR_SIGNALING_PATHWAY | -1.72 | 0.0236 | 0.0192 |
| HEMATOPOIETIC_CELL_LINEAGE | -2.21 | 0.0236 | 0.0192 |
| ANTIGEN_PROCESSING_AND_PRESENTATION | -2.32 | 0.0236 | 0.0192 |
| LEISHMANIA_INFECTION | -2.29 | 0.0236 | 0.0192 |
| VIRAL_MYOCARDITIS | -1.93 | 0.0236 | 0.0192 |
| AUTOIMMUNE_THYROID_DISEASE | -2.07 | 0.0236 | 0.0192 |
| INTESTINAL_IMMUNE_NETWORK_FOR_IGA_PRODUCTION | -2.28 | 0.0236 | 0.0192 |
| GRAFT_VERSUS_HOST_DISEASE | -2.26 | 0.0236 | 0.0192 |
| ALLOGRAFT_REJECTION | -2.05 | 0.0236 | 0.0192 |
| PRIMARY_IMMUNODEFICIENCY | -2.03 | 0.0236 | 0.0192 |
| ASTHMA | -1.94 | 0.0236 | 0.0192 |
| PATHWAYS_IN_CANCER | -1.56 | 0.0326 | 0.0266 |
| AXON_GUIDANCE | -1.63 | 0.0338 | 0.0275 |
| ARACHIDONIC_ACID_METABOLISM | -1.72 | 0.0351 | 0.0286 |
| COMPLEMENT_AND_COAGULATION_CASCADES | -1.74 | 0.0351 | 0.0286 |
| TYPE_I_DIABETES_MELLITUS | -1.90 | 0.0354 | 0.0288 |
| JAK_STAT_SIGNALING_PATHWAY | -1.60 | 0.0411 | 0.0335 |
| LEUKOCYTE_TRANSENDOTHELIAL_MIGRATION | -1.62 | 0.0418 | 0.0341 |
| T_CELL_RECEPTOR_SIGNALING_PATHWAY | -1.61 | 0.0419 | 0.0341 |
| ECM_RECEPTOR_INTERACTION | -1.71 | 0.0422 | 0.0344 |
| VEGF_SIGNALING_PATHWAY | -1.78 | 0.0428 | 0.0349 |
| NOD_LIKE_RECEPTOR_SIGNALING_PATHWAY | -1.78 | 0.0432 | 0.0352 |
| FC_GAMMA_R_MEDIATED_PHAGOCYTOSIS | -1.58 | 0.0492 | 0.0401 |
| DILATED_CARDIOMYOPATHY | -1.62 | 0.0492 | 0.0401 |
