# Supplementary material for: In silico analysis to identify miR-1271-5p/PLCB4 (phospholipase C Beta 4) axis mediated oxaliplatin resistance in metastatic colorectal cancer
Source: Sci Rep. 2023 Mar 16;13:4366. doi: 10.1038/s41598-023-31331-2 (PMC10020571; doi:10.1038/s41598-023-31331-2)
Supplement: Supplementary file 5 — Supplementary Figure 1. [file 41598_2023_31331_MOESM5_ESM.docx]

**Supplementary Figures**

**
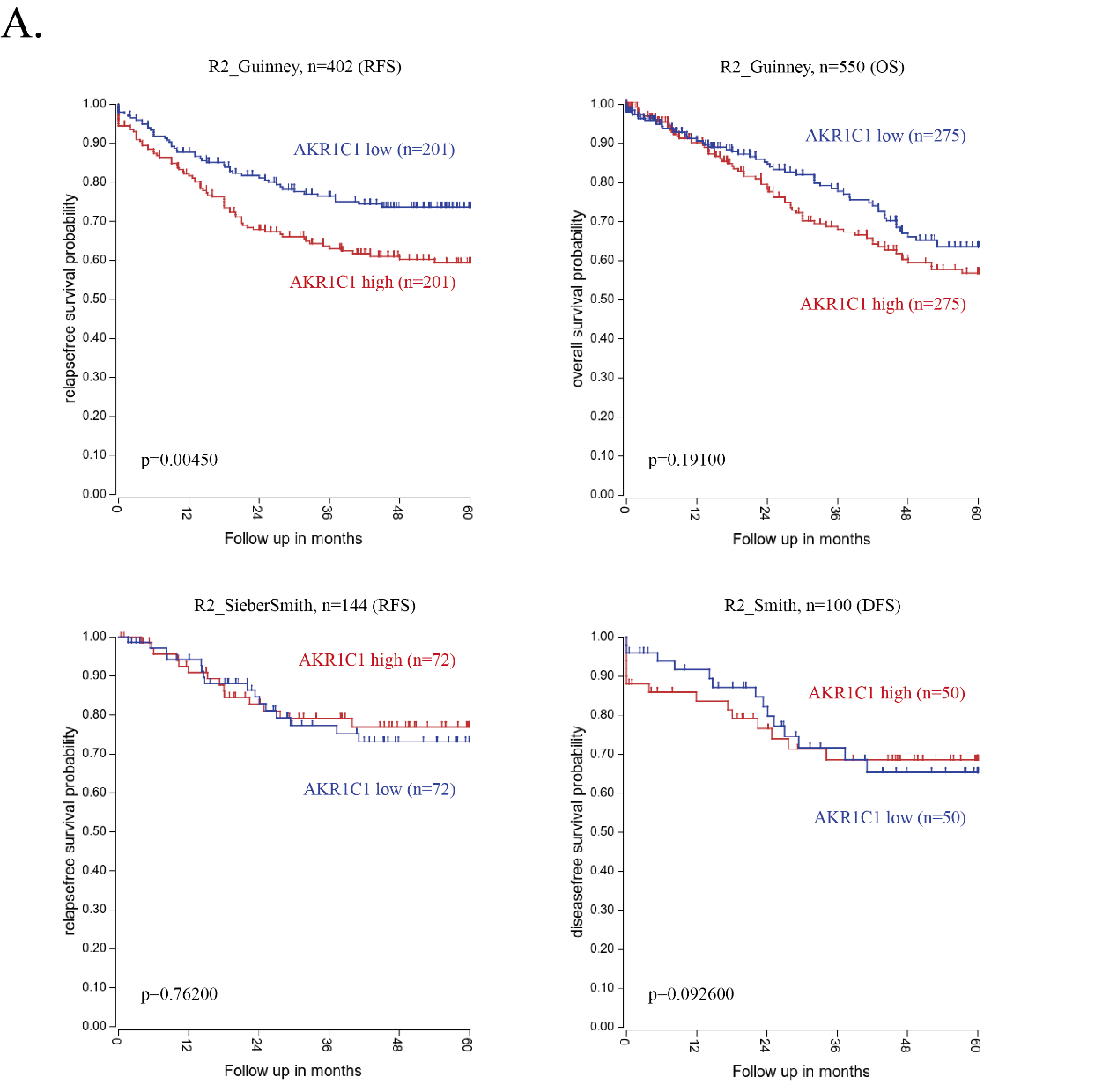
**

**
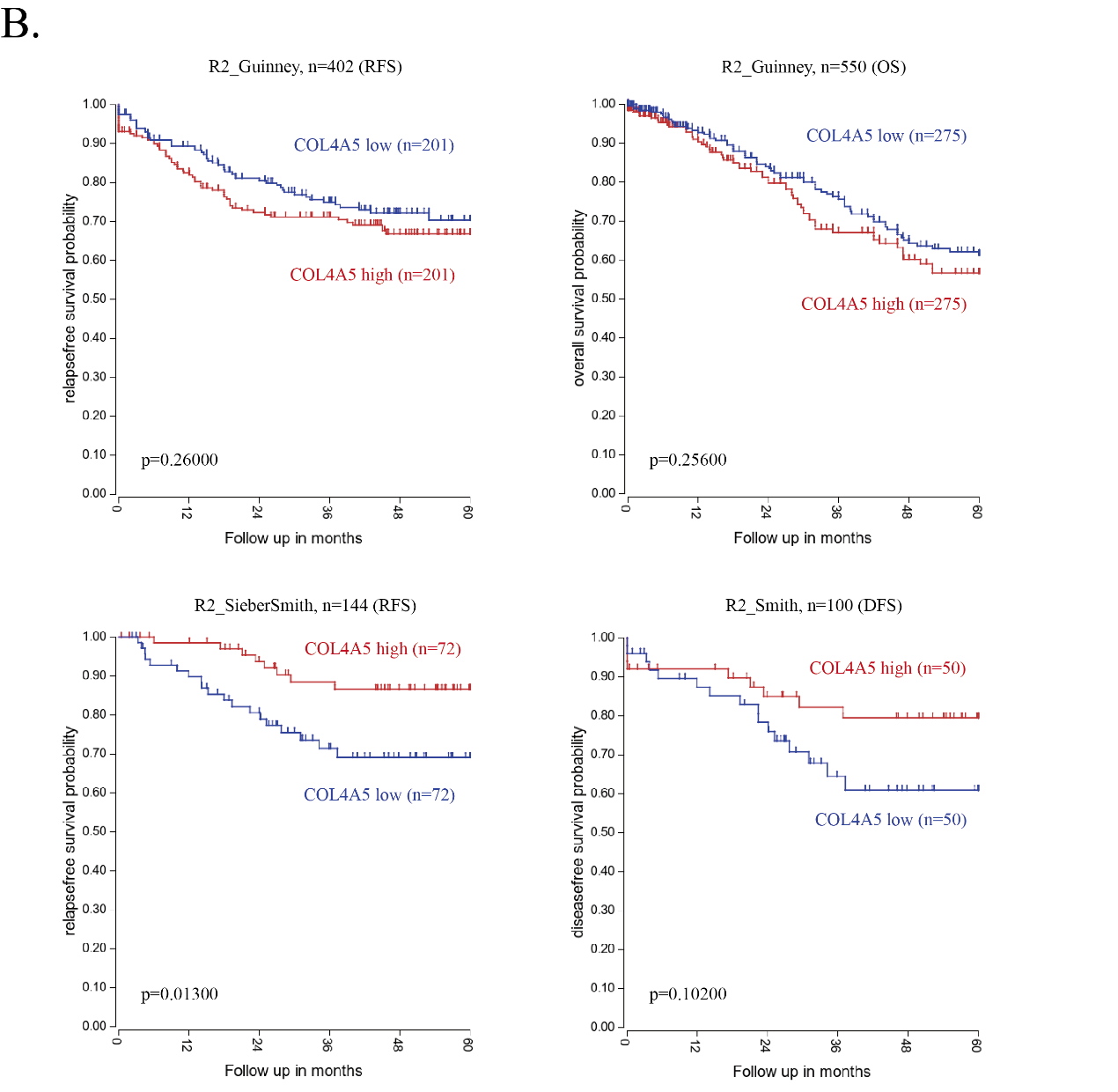
**

**
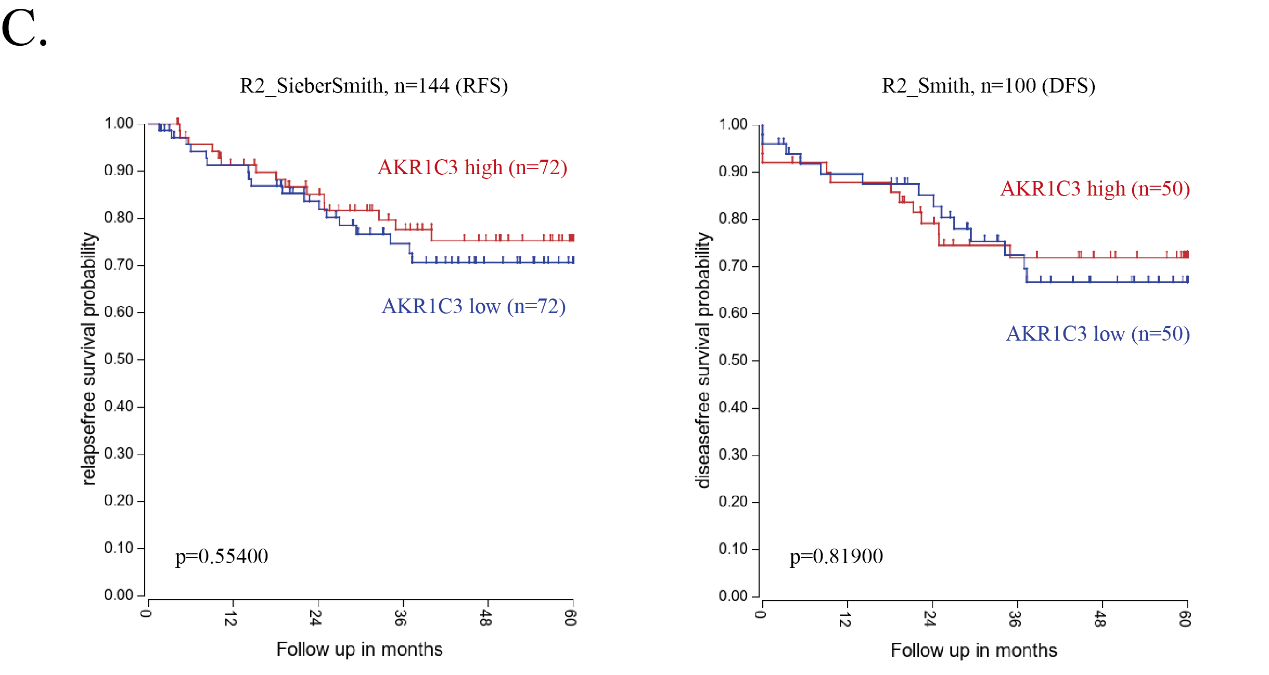
**

**
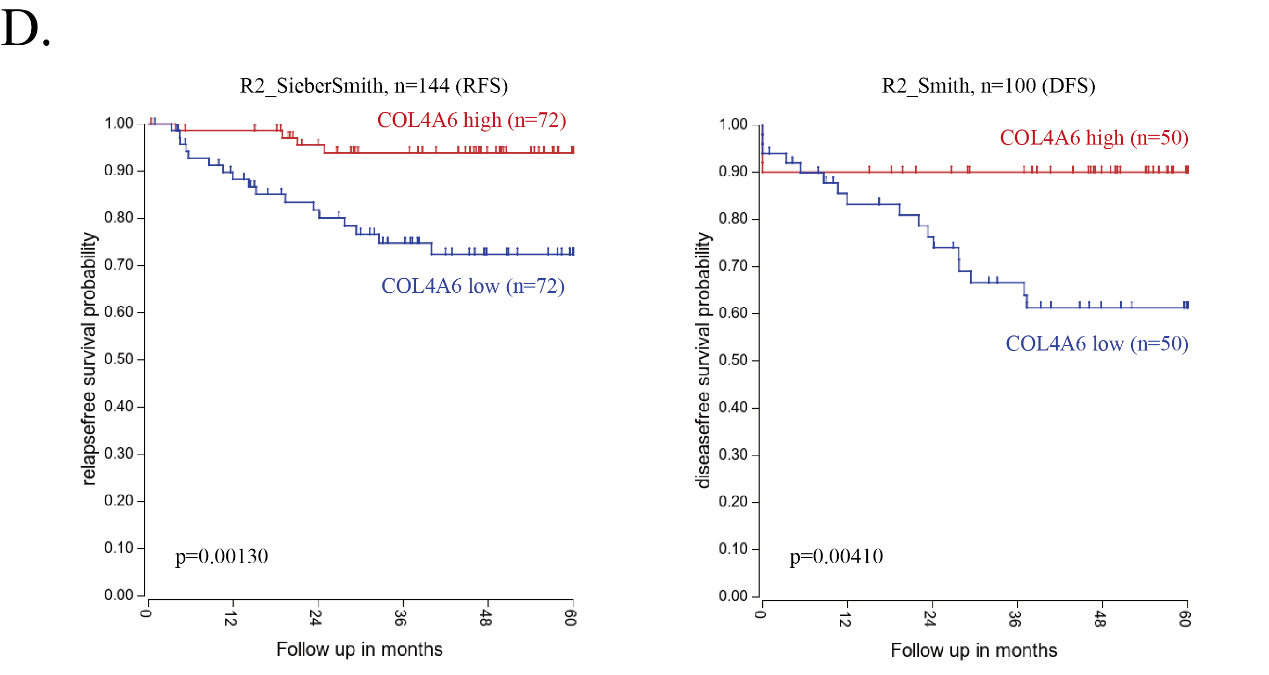
**

**
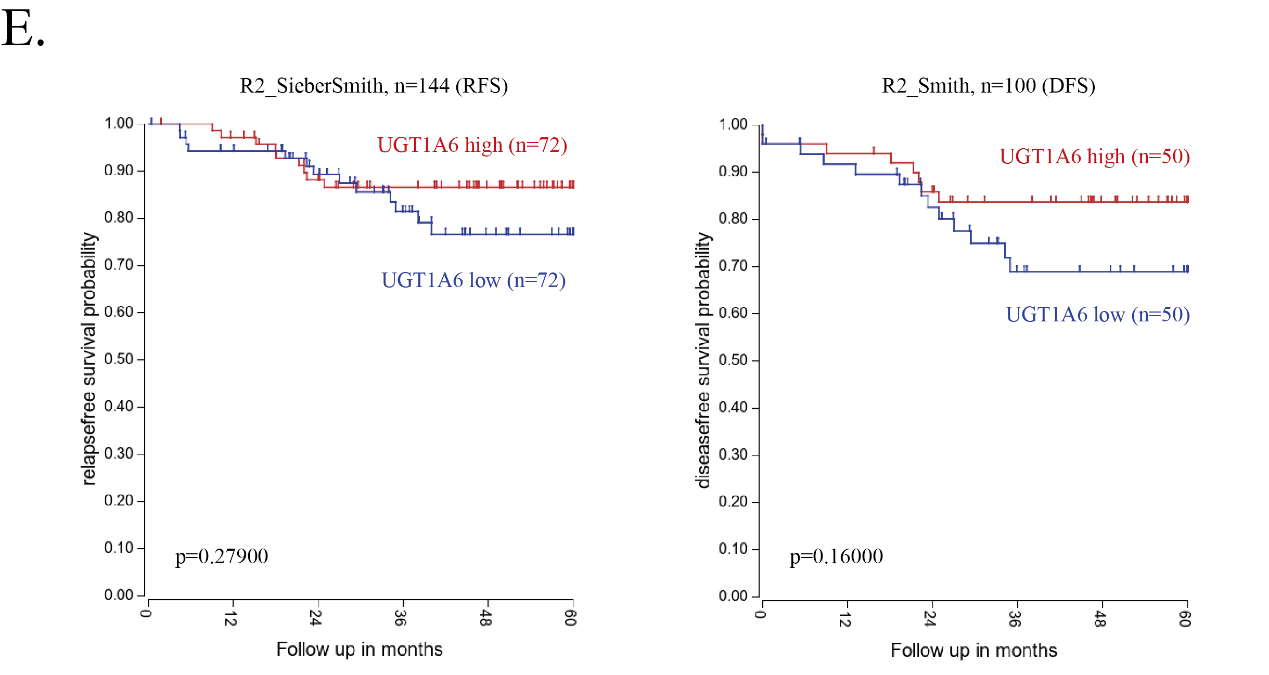
**

**
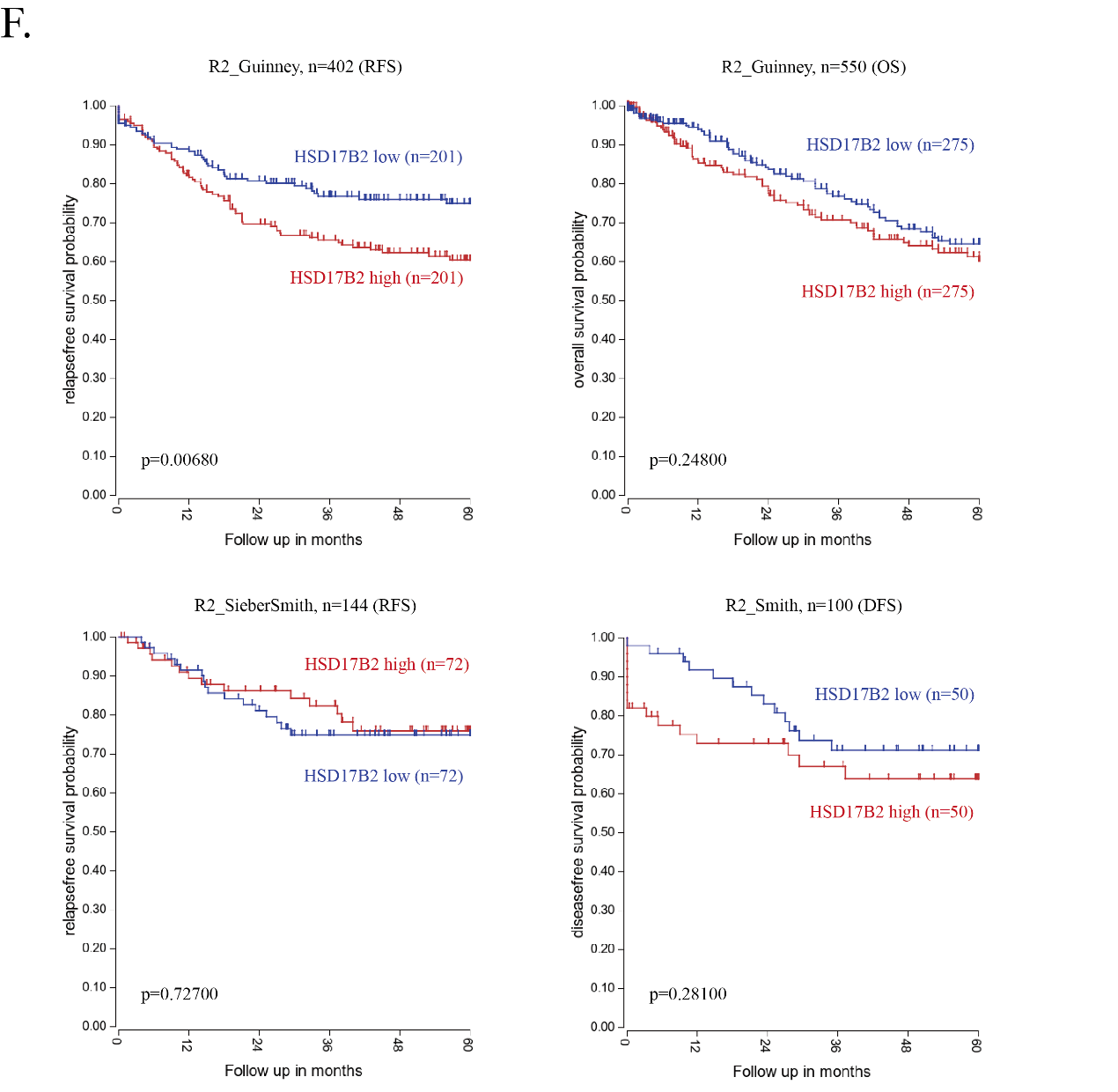
**

**
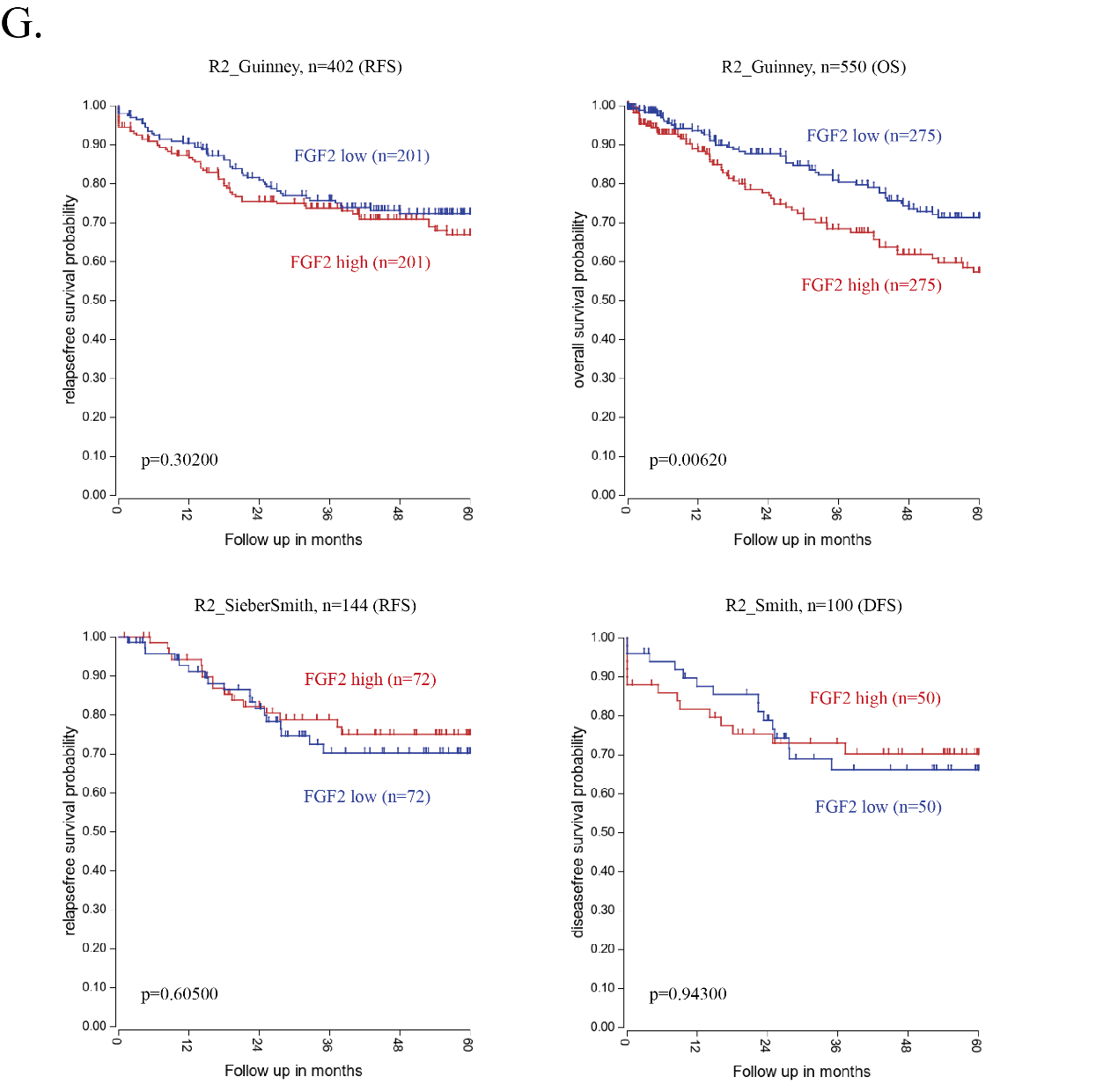
**

**
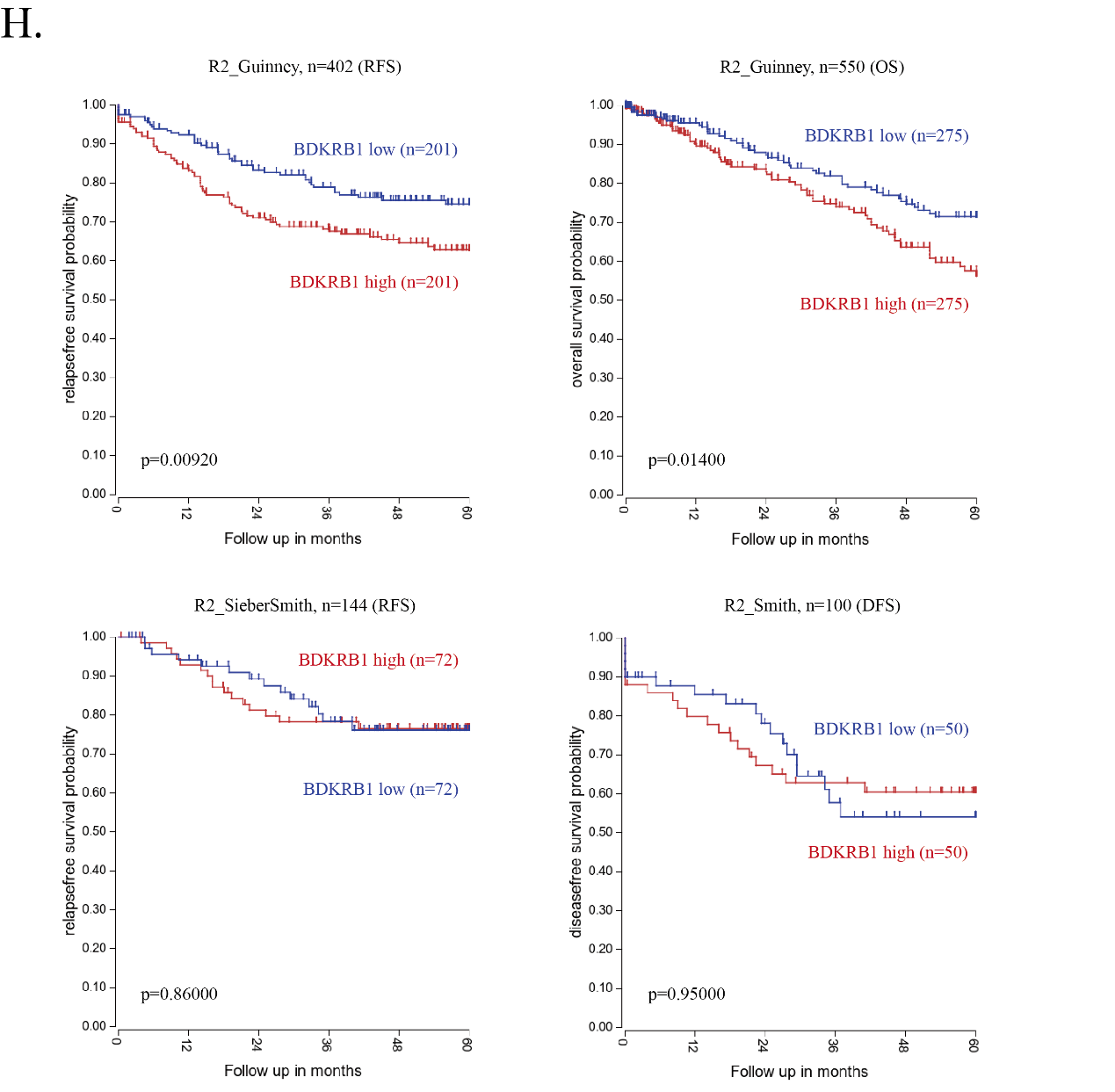
**

**
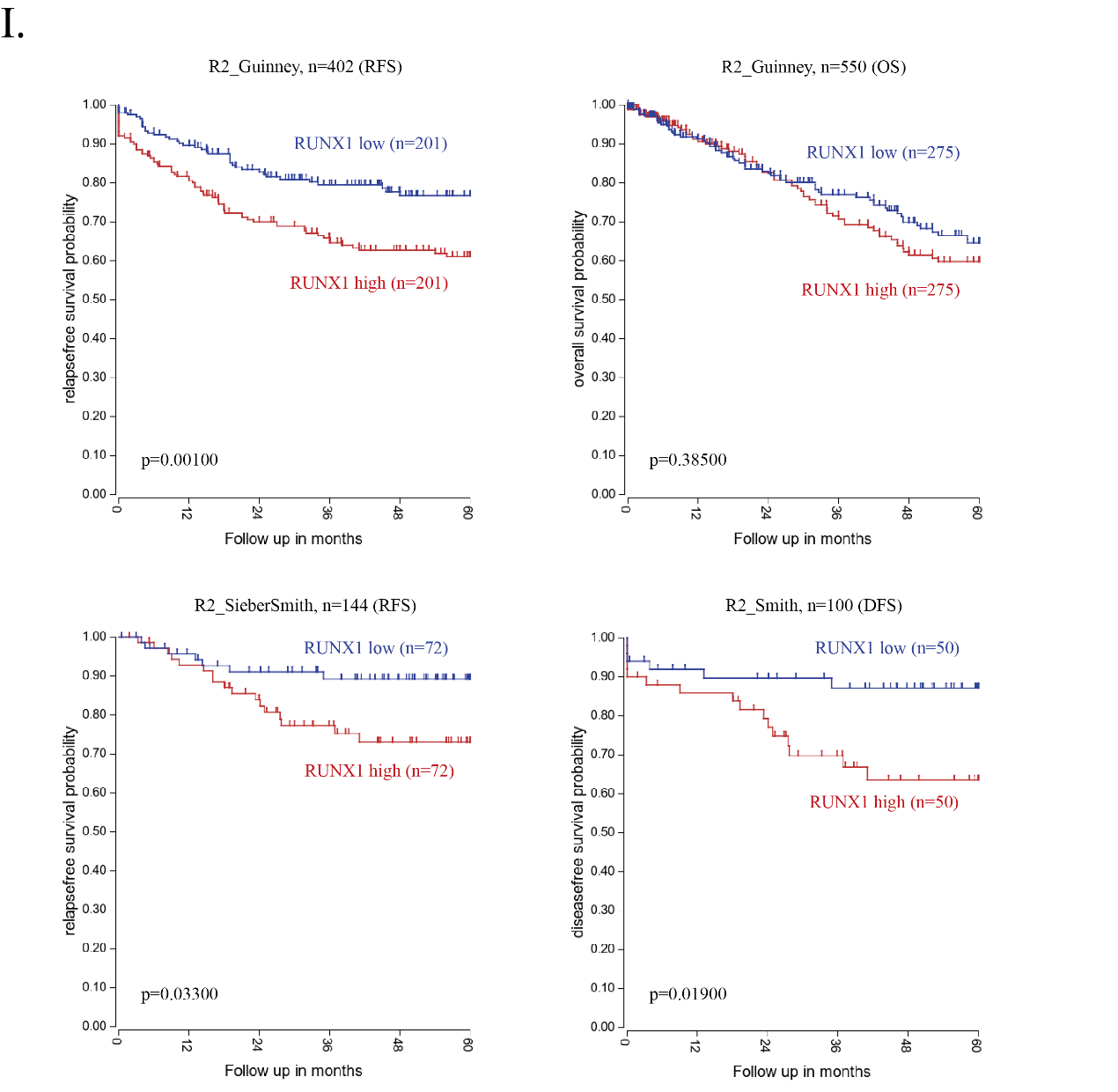
**

**
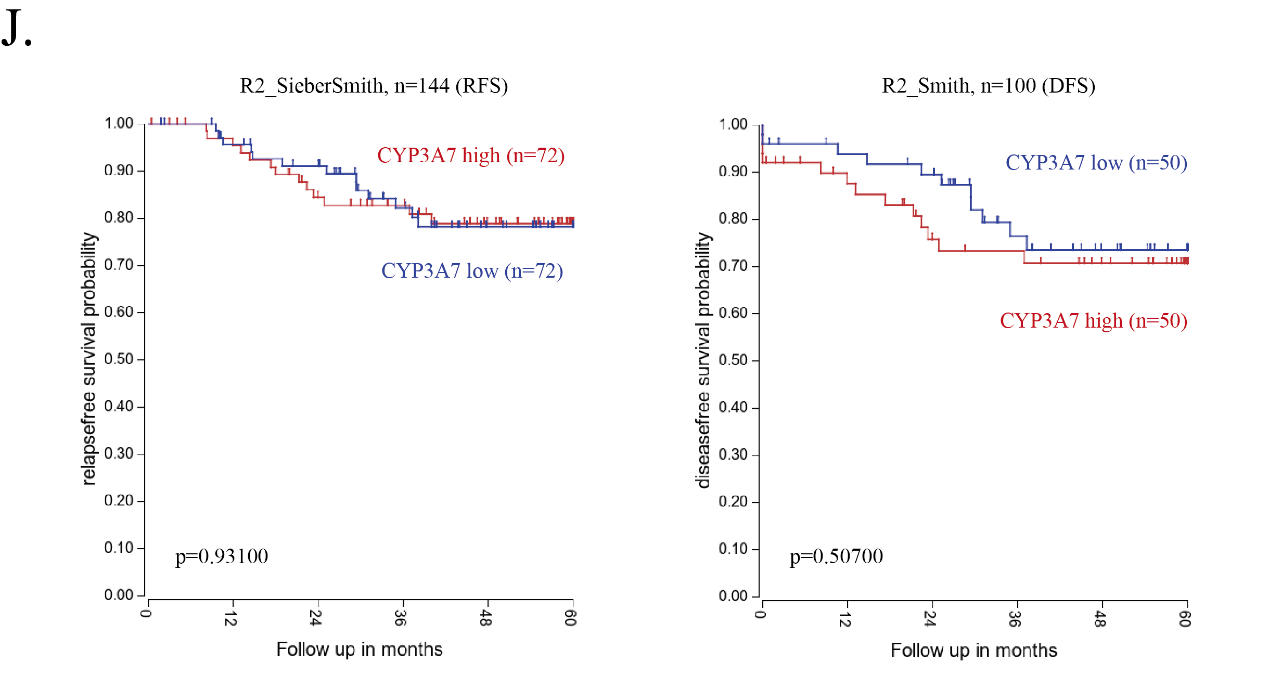
**

**
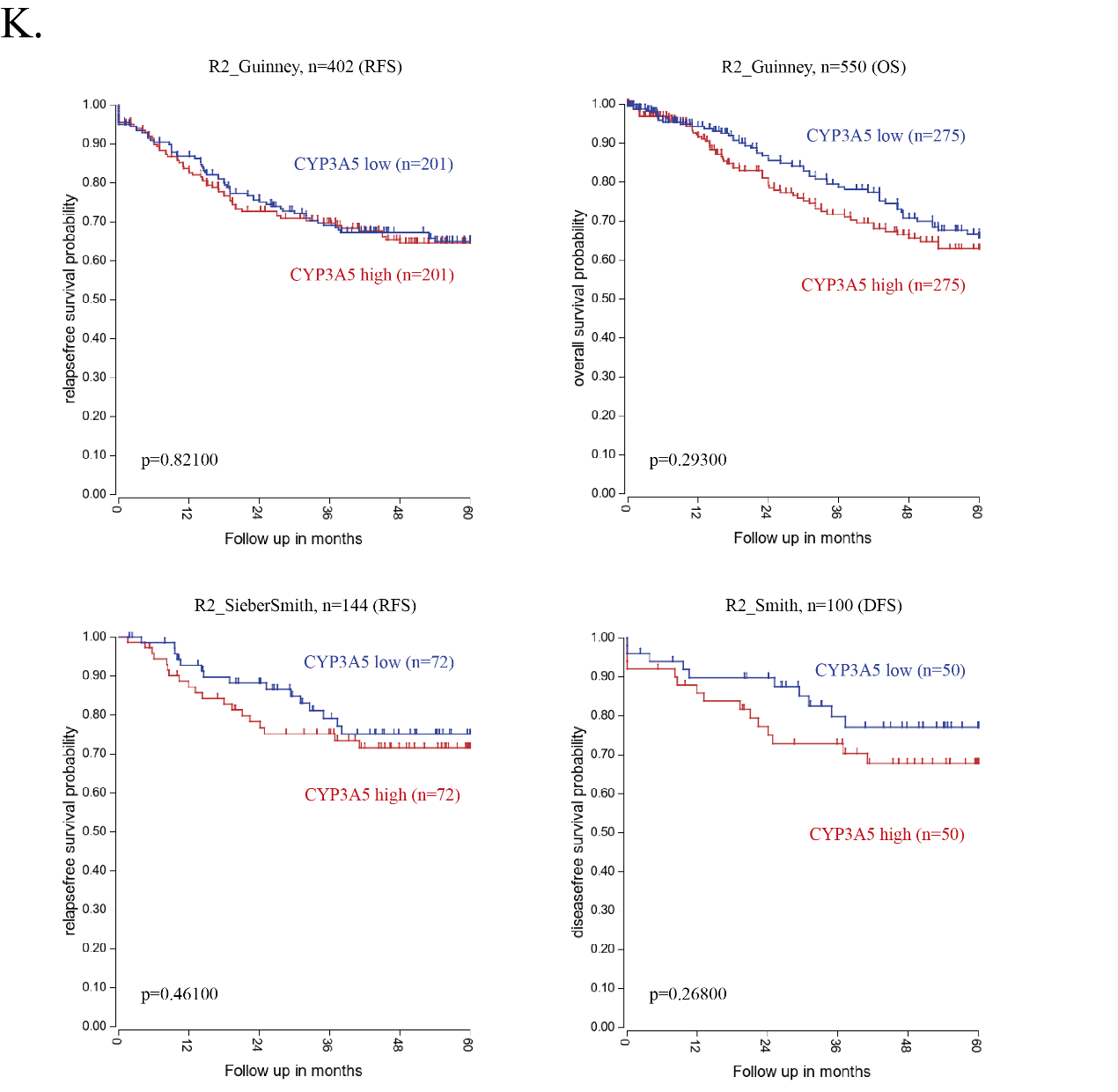
**

**
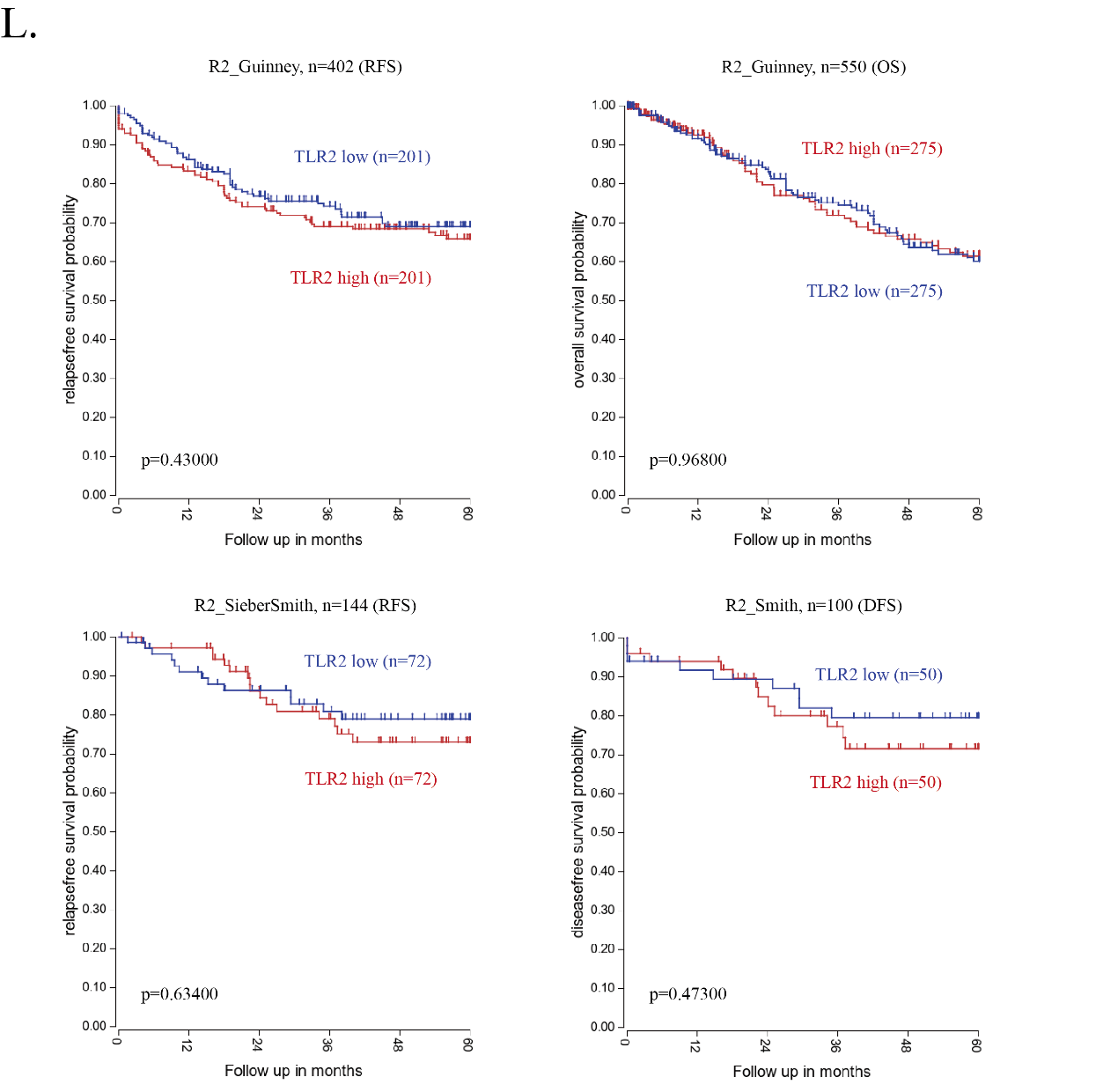
**

**
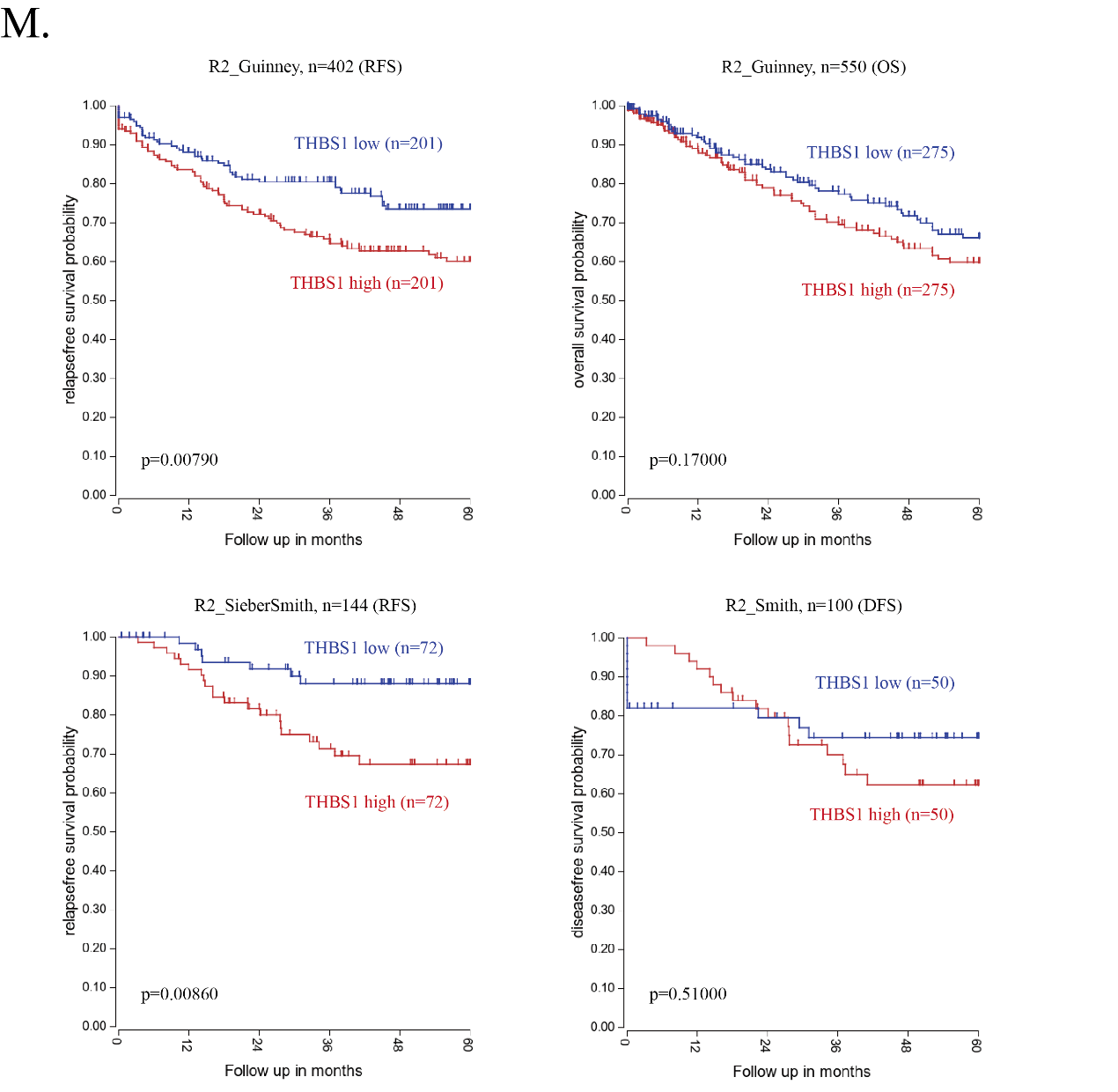
**

**
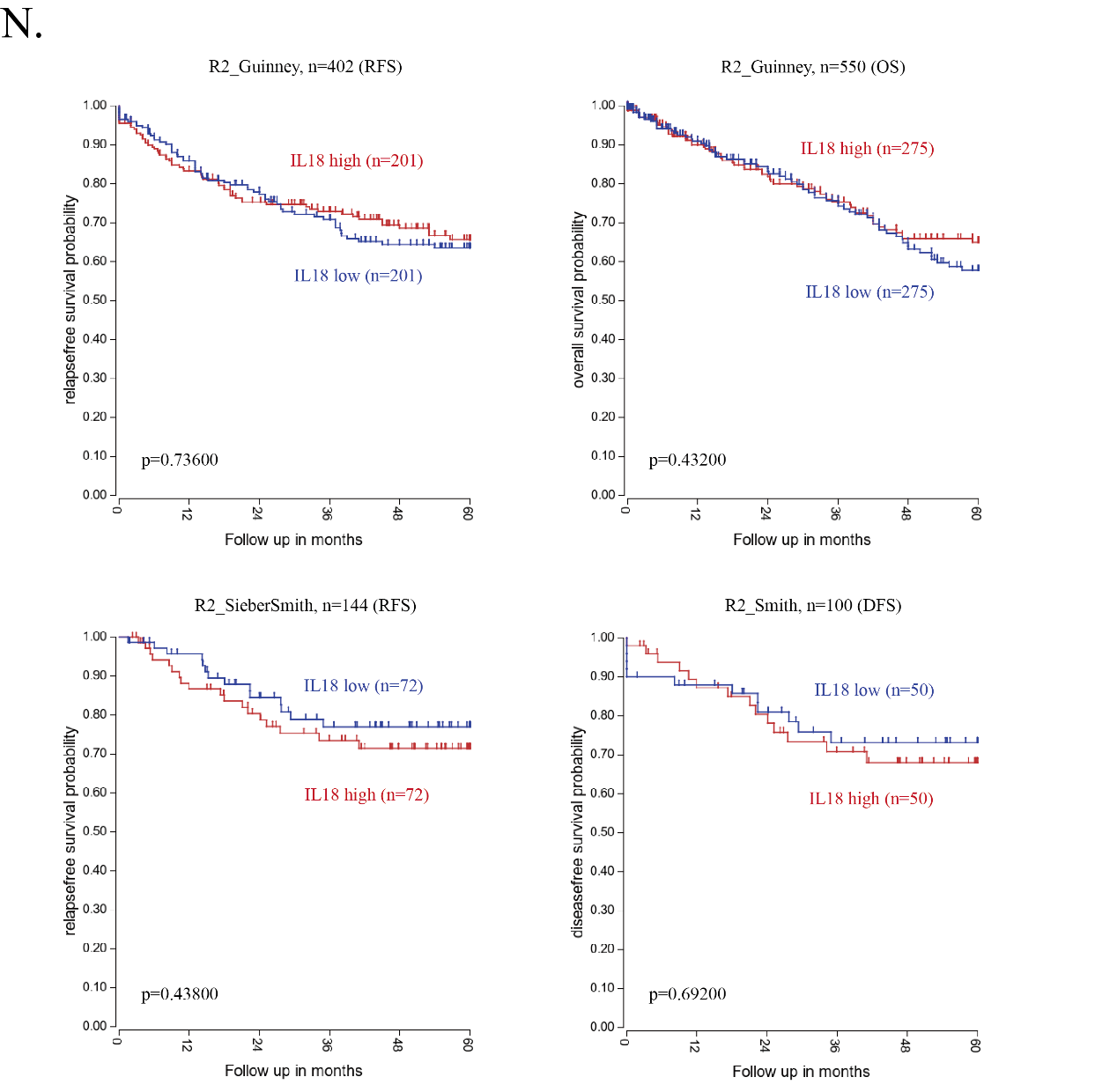
**

**
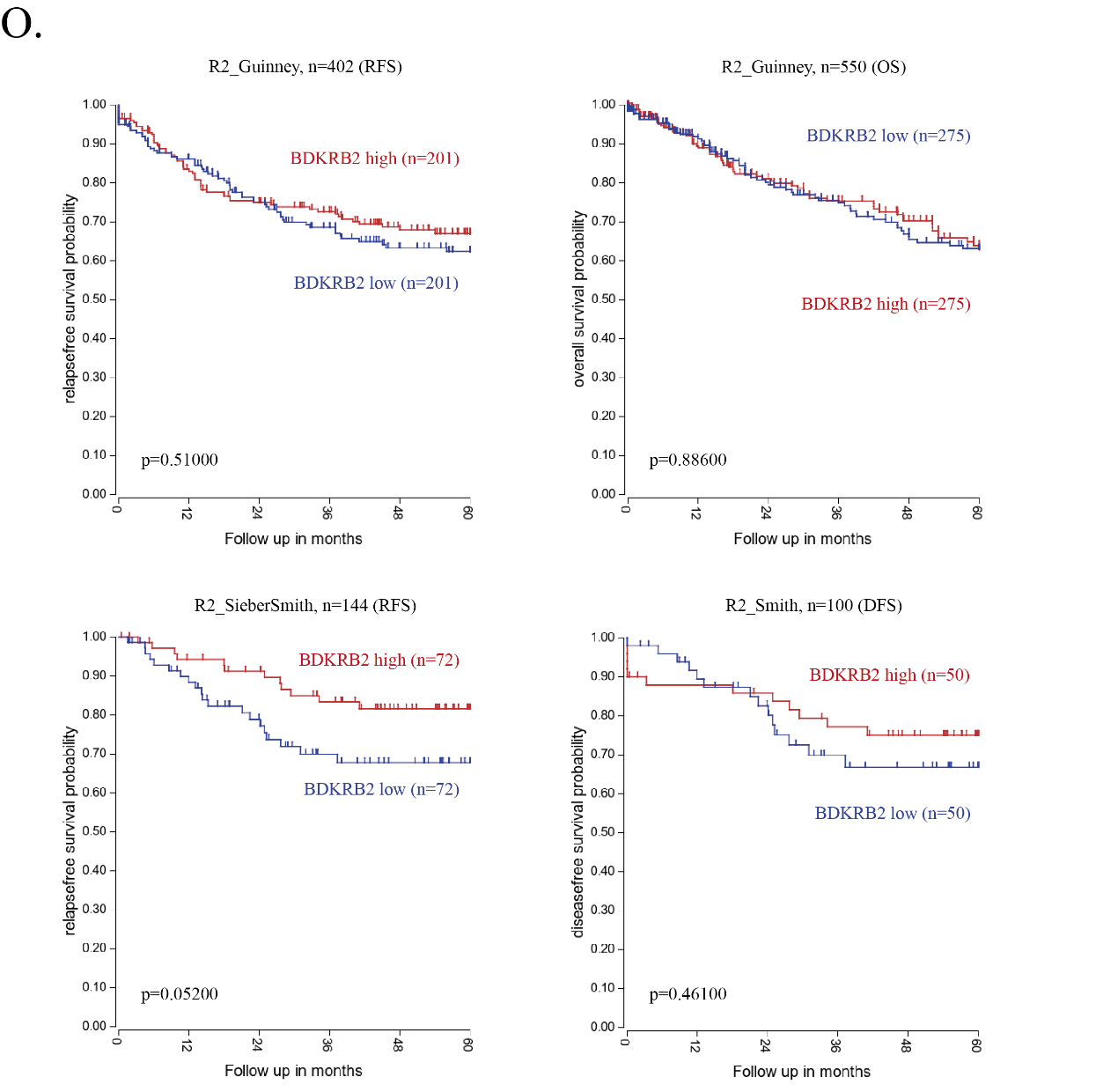
**

**
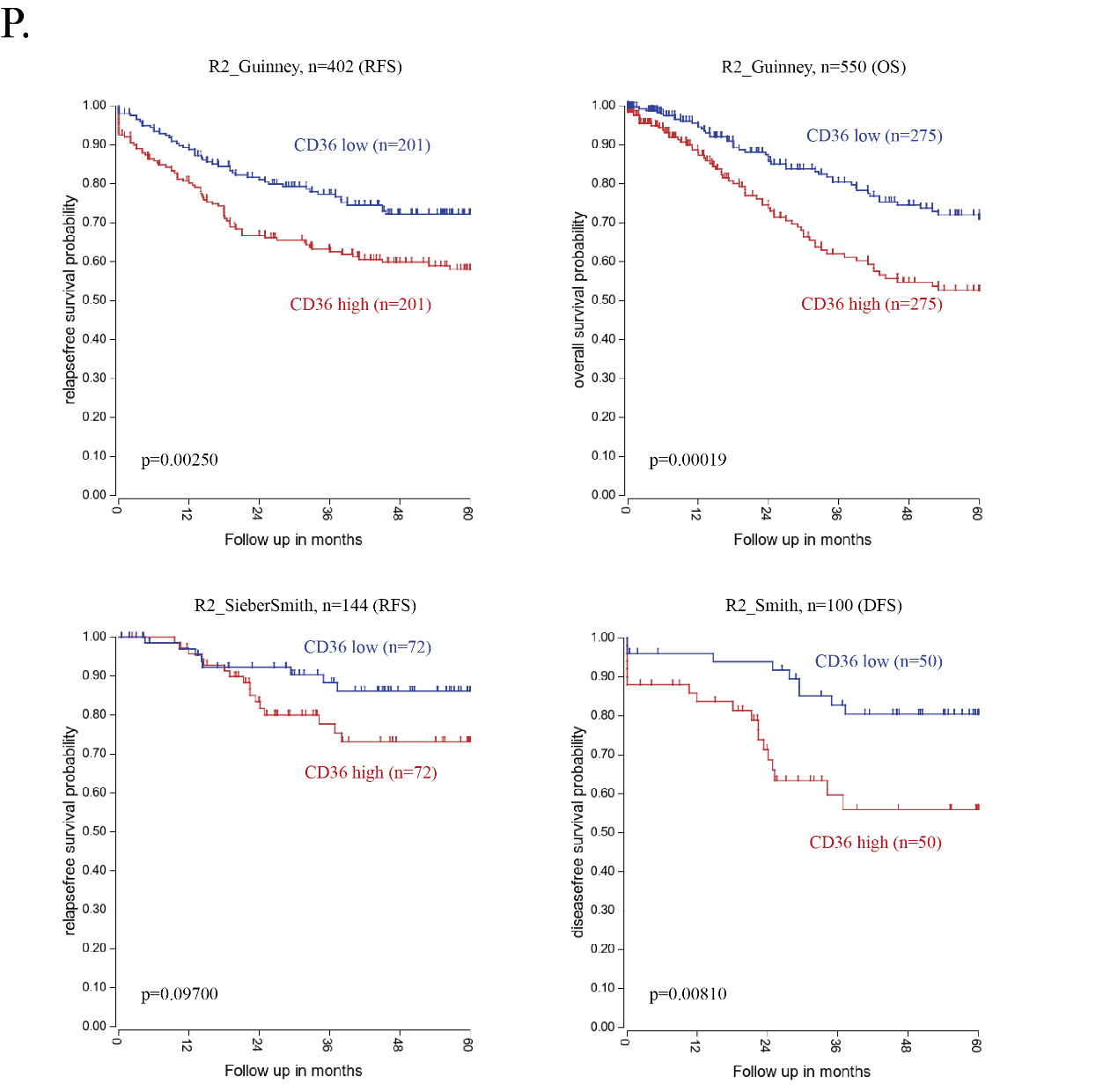
**

**
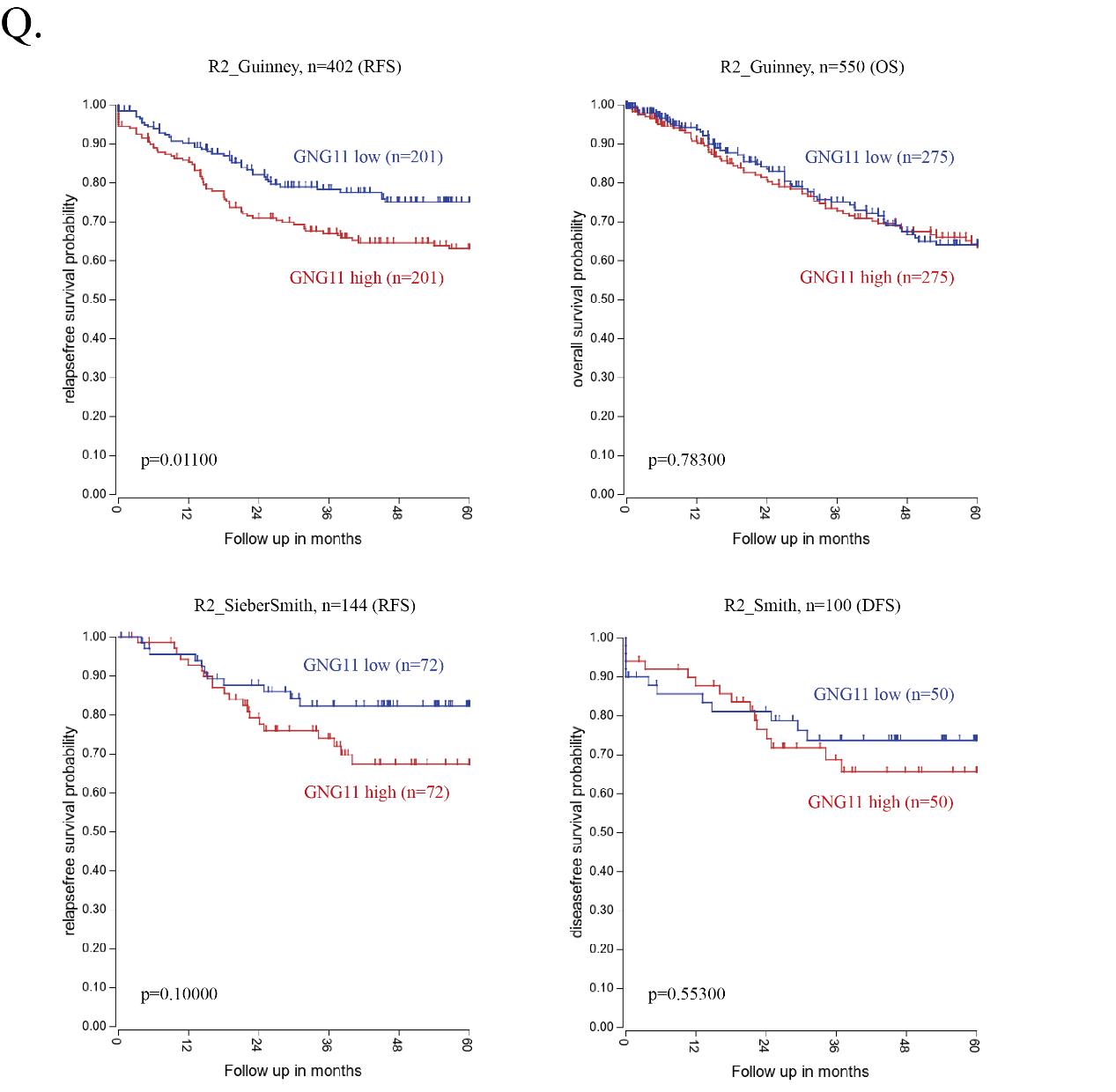
**

**
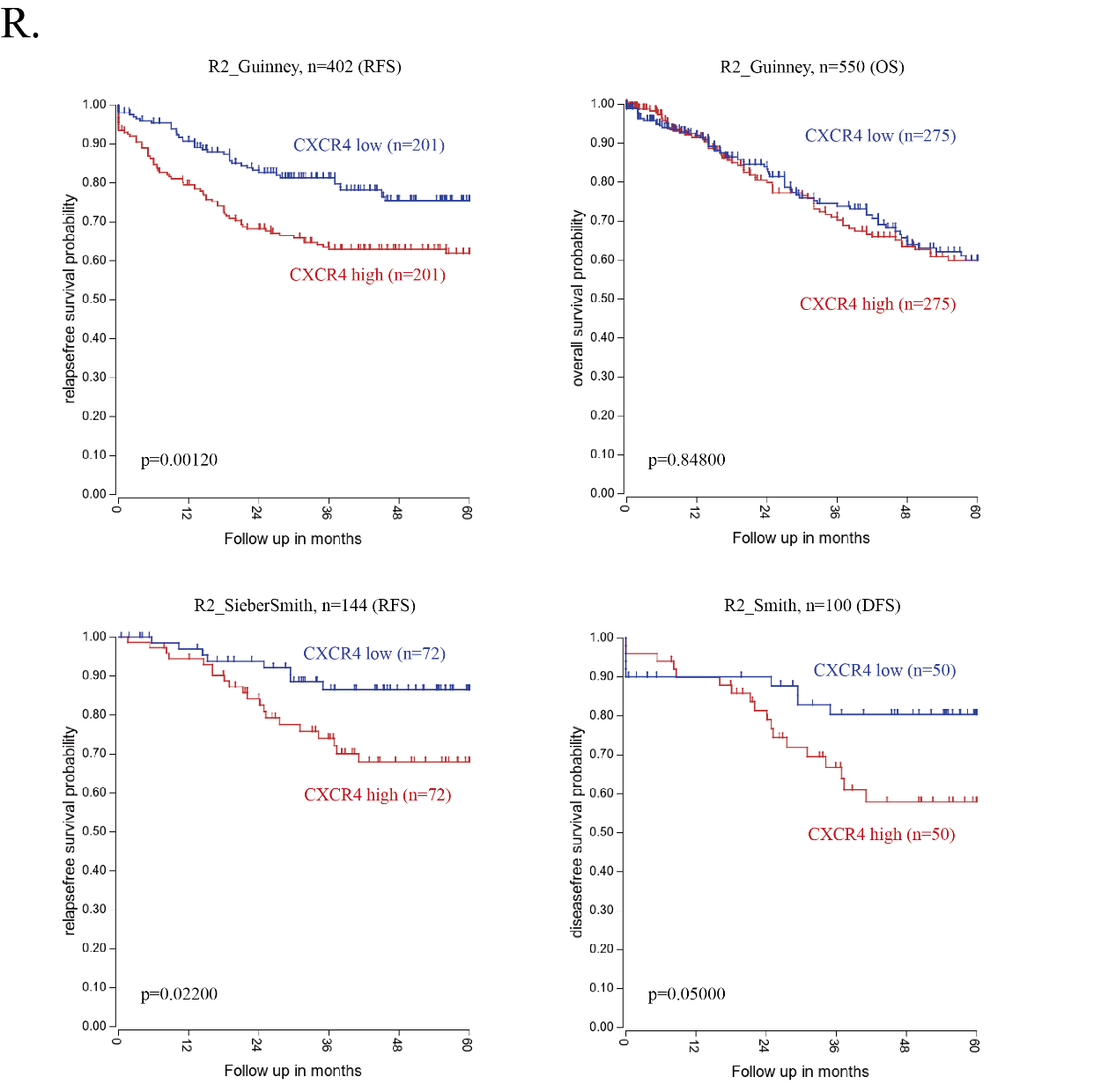
**

**Supplemental Figure 1** Kaplan Meier survival curves presenting the prognostic relationship between high and low expression of key genes by using the R2 platform. Up-regulated genes: (A) AKR1C1 (B) COL4A5 (C) AKR1C3 (D) COL4A6 (E) UGT1A6; down-regulated genes: (F) HSD17B2 (G) FGF2 (H) BDKRB1 (I) RUNX1 (J) CYP3A7 (K) CYP3A5 (L) TLR2 (M) THBS1 (N) IL18 (O) BDKRB2 (P) CD36 (Q) GNG11 (R) CXCR4.
